# A novel regulatory interplay between atypical B_12_ riboswitches and uORF translation in *Mycobacterium tuberculosis*

**DOI:** 10.1093/nar/gkae338

**Published:** 2024-05-06

**Authors:** Terry Kipkorir, Peter Polgar, Declan Barker, Alexandre D’Halluin, Zaynah Patel, Kristine B Arnvig

**Affiliations:** Institute for Structural and Molecular Biology, University College London, Gower Street, WC1E 6BT London, UK; Institute for Structural and Molecular Biology, University College London, Gower Street, WC1E 6BT London, UK; Institute for Structural and Molecular Biology, University College London, Gower Street, WC1E 6BT London, UK; Institute for Structural and Molecular Biology, University College London, Gower Street, WC1E 6BT London, UK; Institute for Structural and Molecular Biology, University College London, Gower Street, WC1E 6BT London, UK; Institute for Structural and Molecular Biology, University College London, Gower Street, WC1E 6BT London, UK

## Abstract

Vitamin B_12_ is an essential cofactor in all domains of life and B_12_-sensing riboswitches are some of the most widely distributed riboswitches. *Mycobacterium tuberculosis*, the causative agent of tuberculosis, harbours two B_12_-sensing riboswitches. One controls expression of *metE*, encoding a B_12_-independent methionine synthase, the other controls expression of *ppe2* of uncertain function. Here, we analysed ligand sensing, secondary structure and gene expression control of the *metE* and *ppe2* riboswitches. Our results provide the first evidence of B_12_ binding by these riboswitches and show that they exhibit different preferences for individual isoforms of B_12_, use distinct regulatory and structural elements and act as translational OFF switches. Based on our results, we propose that the *ppe2* switch represents a new variant of Class IIb B_12_-sensing riboswitches. Moreover, we have identified short translated open reading frames (uORFs) upstream of *metE* and *ppe2*, which modulate the expression of their downstream genes. Translation of the *metE* uORF suppresses MetE expression, while translation of the *ppe2* uORF is essential for PPE2 expression. Our findings reveal an unexpected regulatory interplay between B_12_-sensing riboswitches and the translational machinery, highlighting a new level of *cis*-regulatory complexity in *M. tuberculosis*. Attention to such mechanisms will be critical in designing next-level intervention strategies.

## Graphical Abstract

**Graphical Abstract:**
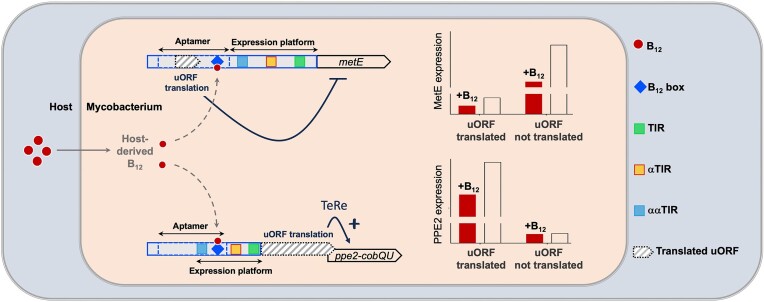

## Introduction

RNA leaders preceding coding sequences in mRNAs have gained interest as hubs for gene expression control (1–4). These include riboswitches, which are highly structured *cis*-regulatory RNAs that sense and bind specific metabolites such as enzyme cofactors, amino acids, or nucleotides, to affect the expression of genes under their control (5–7). Typically, the regulated genes have a direct relationship with the corresponding riboswitch ligand, whereby the encoded gene product is involved in the *de novo* biosynthesis of the ligand or its transport (8,9). Riboswitches regulate gene expression via the interaction between two RNA domains: the aptamer and the expression platform. Aptamers are highly conserved and form a unique 3D structure for ligand-binding. Expression platforms execute gene regulation by adopting mutually exclusive secondary structures depending on the ligand binding status of the aptamer (3,4). Although the mechanisms of individual riboswitches can vary, the gene expression outcome is either permissive (‘ON’ switch) or non-permissive (‘OFF’ switch), primarily due to changes in transcription termination or translation initiation or both (10–12). Termination of transcription can be intrinsic or Rho-dependent, the latter being the dominant mechanism genome-wide in mycobacteria (13). Riboswitch-mediated translational control typically involves ligand-dependent occlusion of the translation initiation region (TIR) by a complementary anti-TIR (αTIR) sequence, which, in turn, may be sequestered by an anti-anti-TIR (ααTIR) sequence (12). Inhibition of translation may in turn facilitate downstream Rho-dependent transcription termination, as Rho-binding (RUT) sites become exposed on the RNA (14–17). As intrinsic terminators are rare in mycobacteria, mycobacterial riboswitches are presumably mostly translational and/or Rho-dependent (4,13).

**Figure 1.**
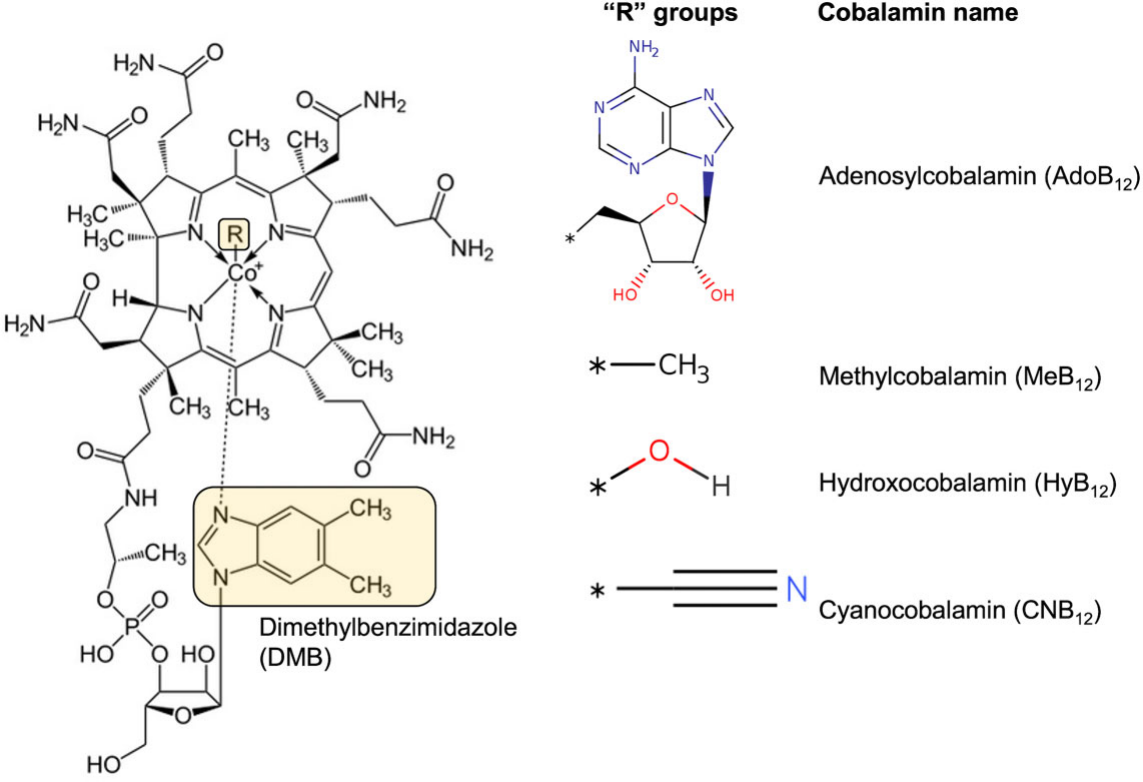
Chemical structure of cobalamin. The different functional groups (‘R’ groups) that can occupy the β-axial positions are shown to the right of the structure. Dimethylbenzimidazole (DMB) is highlighted on the structure.

Numerous riboswitch families regulating a wide range of cellular processes have been discovered in the last two decades (18–27). One of the most widespread of these are riboswitches that bind coenzyme B_12_ (cobalamin), regulating genes involved in the biosynthesis, transport or utilization of this cofactor (26,28–30). B_12_ is a complex organometallic compound whose structure is based on a corrin ring containing a central cobalt ion coordinated by lower (α-axial) and upper (β-axial) ligands (Figure 1) (31). The α-axial ligand is typically dimethylbenzimidazole (DMB) (32), while the β-axial ligand can be one of several functional ‘R’ groups. Adenosyl (Ado-), methyl (Me-), hydroxy (Hy-) and cyano (CN-) groups are the most common (Figure 1) (31), and adenosylcobalamin (AdoB_12_), methylcobalamin (MeB_12_), and hydroxocobalamin (HyB_12_) are naturally occurring B_12_ isoforms.

*Mycobacterium tuberculosis*, the aetiological agent of tuberculosis (33,34), as well as *M. africanum* and animal adapted lineages, lack the ability to synthesize B_12_ (35,36), linked to the deletion of *cobF*, encoding a precorrin-6A synthase, and N-terminus truncation of *cobL*, encoding a decarboxylating precorrin-6Y C (5,15)-methyltransferase, as both are involved in the *de novo* B_12_ biosynthesis pathway (37–39). Recent evidence suggests that the ablation of *de novo* B_12_ biosynthesis in these organisms shaped their evolution as pathogens capable of systemic infections (40). Still, *M. tuberculosis* encodes several B_12_-dependent enzymes, suggesting that the pathogen utilizes host-derived B_12_, although these enzymes and their pathways are found alongside alternative B_12_-independent counterparts in *M. tuberculosis*. This is true for the conversion of ribonucleotides to deoxyribonucleotides (41,42), for the degradation of propionate (41,42) and for the biosynthesis of methionine from homocysteine (41,42). Methionine synthesis and propionate degradation are both B_12_-dependent pathways in humans (43). In addition, a role for the host-derived B_12_ in virulence and as a signalling molecule in the cross-talk between the host and *M. tuberculosis* has been suggested (40–42).

B_12_ has been shown to regulate expression of *metE* via B_12_-sensing riboswitches in *M. tuberculosis* and in the saprophytic mycobacterial model, *Mycobacterium smegmatis*, by suppressing *metE* mRNA levels (44,45). A second, homologous B_12_ riboswitch occurs in *M. tuberculosis* upstream of a potential tricistronic operon comprising *ppe2*, *cobQ* and *cobU*. PPE2 is a member of the large, *Mycobacterium*-specific PE/PPE protein family associated with host-pathogen interactions and virulence (46–48) and is suspected to be involved in cobalt transport (49). PPE2 has also been suggested to suppress nitric oxide production in host macrophages and hence affect the host immune response (50–52). CobQ and CobU are relics of the disrupted B_12_ biosynthesis pathway (49).

Biochemical and structural studies of B_12_ riboswitches in different bacteria have provided detailed insights into conserved features such as the central, ligand-binding four-way junction, the B_12_ box and the ‘kissing loop’ (KL). Despite the conservation of these features, B_12_ riboswitches exhibit differential selectivity to individual B_12_ isoforms and variations in their peripheral elements (53,54). These characteristics form the basis for their division into Class I, IIa & IIb switches. Class I and IIb riboswitches selectively bind AdoB_12_, whereas Class IIa riboswitches show preferential binding to the slightly smaller MeB_12_ and HyB_12_. Other B_12_ riboswitches displaying promiscuous binding to a broad range of corrinoids have also been reported (49).

Recently, it has been proposed that codons and/or peptides arising from the translation of upstream open reading frames (uORFs) occurring within gene leaders can either positively or negatively impact the expression level of the downstream ORF (13,55–57). For example, translating ribosomes present on uORFs can modulate the expression of the downstream gene, which may involve translation coupling between ORFs (58,59). How riboswitch-associated uORFs might affect gene expression has not yet been addressed.

In the current study, we analyse ligand binding, riboswitch architecture, and control mechanisms of the *metE* and *ppe2* riboswitches from *M. tuberculosis*. We present the first evidence of binding of B_12_ to these elements and functional validation of the *ppe2* riboswitch as an ‘OFF switch.’ We found that the two riboswitches exhibit differential binding of B_12_ isoforms and involve distinct structures in their expression platforms to execute B_12_-dependent control. On this basis, we propose that the *ppe2* switch represents a new variant of Class IIb switches whereas the *metE* switch presents as a Class I member. Moreover, we show that translation of uORFs in the leaders of *metE* and *ppe2* substantially alters the expression of their respective downstream coding regions. Interestingly, translation of the *metE* uORF suppresses MetE expression whereas translation of the *ppe2* uORF is essential for PPE2 expression. In the latter case, we found evidence of termination-reinitiation (TeRe) (59) in LacZ reporter constructs, resulting in separate uORF and LacZ proteins as well as uORF stop codon suppression, leading to a frameshifted uORF-PPE2 fusion protein. The unexpected variation and complexity of *M. tuberculosis* B_12_ riboswitch-regulation expands our current understanding of such elements and reveals new intricacies of translational control in this pathogen.

## Materials and methods

### Strains and culture conditions

The *M. smegmatis* Δ*cobK* mutant was a gift from Professor Digby Warner. *E. coli* cells were grown either in Lysogeny Broth (LB) or on LB agar, with 250 μg/ml hygromycin, where appropriate. *M. smegmatis* cultures were grown either on Middlebrook 7H11 agar or Middlebrook 7H9 broth (Sigma-Aldrich) supplemented with glucose–salt solution (0.085% NaCl and 0.2% glucose). The *M. tuberculosis* H37Rv strain was cultured either in 7H9 broth supplemented with 10% ADC (Remel) or on 7H11 agar supplemented with 10% Middlebrook OADC (Becton-Dickinson). Cloning was performed in *Escherichia coli* DH5α electrocompetent cells (New England Biolabs). Plasmids were extracted with Qiagen miniprep kits (Qiagen GmbH) and verified via Sanger sequencing (Source Biosciences). Transformation in mycobacteria was performed by electroporation using the exponential protocol settings (2.5 kV; 25 μF; 1000 Ω). For antibiotics selection and blue–white screening of mycobacteria, 7H11 plates contained 50 μg/ml hygromycin and 50 μM 5-bromo-4-chloro-3-indolyl-β-d-galactopyranoside (X-gal) (Thermo Scientific). Unless specified, *M. tuberculosis* H37Rv culture was supplemented with 10 μM exogenous adenosylcobalamin (Thermo Scientific). Methylcobalamin, and hydroxocobalamin were purchased from Cambridge Bioscience whereas cyanocobalamin was obtained from Sigma-Aldrich.

### Oligonucleotides, plasmids and cloning

Oligos and plasmids used in this study are listed in Supplementary Table S1. DNA oligos longer than 100 bp were purchased as geneBlocks fragments from Integrated DNA Technologies. Other oligos and primers were purchased from Thermo Fisher Scientific. Reporter constructs were generated by inserting target sequences using either Gibson assembly or restriction cloning between the HindIII and NcoI sites of pIRaTE2020 (13), to produce in-frame translational fusions with LacZ. The 5′ edges of *umetE’-lacZ* and *umetE4’-lacZ* fusions were at +87 nt and +159 nt, respectively, relative to the TSS (+1 nt) of the *metE* leader; the 5′ boundaries of other *lacZ* fusions are specified in the relevant sections of the manuscript. Nucleotide substitution or deletion mutations were designed on the NEBaseChanger online tool (https://nebasechanger.neb.com/) and TOPO-cloned using the Q5 site-directed mutagenesis kit (New England Biolabs) according to the standard protocol.

### RNA extraction and northern blot analysis

*M. tuberculosis* H37Rv cultures were rapidly chilled by directly mixing with ice and pelleted by centrifugation. RNA was isolated using the RNAPro Blue kit (MP Biomedicals) according to the manufacturer's protocol. RNA concentration and quality were evaluated on a Nanodrop 2000 spectrophotometer (Thermo Scientific). For northern blot analysis, 10 μg total RNA was separated on denaturing 8% polyacrylamide gel and transferred on a blotting paper for detection, as previously described (60). For horizontal agarose northerns, RNA was separated on 1% agarose and transferred to blotting paper according to the NorthernMax™ Gly kit (Invitrogen). RNA probes were synthesized using the mirVana miRNA probe synthesis kit (Ambion) and radiolabelled using 133 nM ^32^P α-UTP (3000 Ci/mmol; Hartmann Analytic GmbH), with unlabelled UTP added to achieve a final concentration of 3 μM. RNA fragment signals were developed on radiosensitive screens and visualized on a Typhoon FLA 9500 phosphorimager (GE Healthcare).

### Quantitative real-time PCR

The RNA used for quantitative real-time PCR (qRT-PCR) was isolated from cultures supplemented with 10 μg/ml exogenous AdoB_12_, as reported by others (35,44). qRT-PCR was performed on the QuantStudio 6 real-time PCR system (Applied Biosystems) using cDNA synthesized from 0.5 μg DNase-treated RNA using the Superscript IV Reverse Transcriptase kit (Invitrogen) and random hexamers. Each 20-μl PCR reaction contained 1 × Fast SYBR Green Master Mix (Applied Biosystems), 200 nM forward and reverse primers and 5 μl of 100 × diluted cDNA or *M. tuberculosis* genomic DNA standards. The TSS-proximal amplicon (5′ amplicon) in the *metE* leader covered the section between + 5 nt and + 117 nt relative to the *metE* TSS (genomic coordinate 1261711) (61), while the further downstream leader amplicon extended from +245 nt to +364 nt, relative to the *metE* TSS. The primers for the *metE* coding amplicon amplified +720 nt to +825 nt relative to the *metE* TSS. The *ppe2* leader amplicon extended from +200 nt to +314 nt relative to the *ppe2* TSS (genomic coordinate 309839) (61), while the *ppe2* coding amplicon extended from +1007 nt to +1117 nt relative to the *ppe2* TSS. The *ppe2-cobQ* junction amplicon extended from +1861 nt to +2118 nt relative to the *ppe2* TSS. Relative gene expression was determined as a ratio of the level of target mRNA to that of 16S rRNA. All data were graphed and analysed using GraphPad Prism software for Mac OS, version 10.0 (www.graphpad.com).

### Beta-galactosidase (β-gal) activity assay

Protein expression levels were assessed using the beta-galactosidase (β-gal) activity assay as previously described (60). Briefly, 10-ml cell cultures were centrifuged, and the cell pellet washed thrice in Z-buffer (60 mM Na_2_HPO_4_, 40 mM NaH_2_PO_4_, 10 mM KCl, 1mM MgSO_4_) prior to one round of lysis in a FastPrep bio-pulveriser (MP Biomedicals) at speed = 6.5 and time = 30 s. The protein concentration in the cell lysate was determined using the BCA kit (Thermo Scientific) according to the manufacturer's instructions. The level of *lacZ* expression was calculated in Miller units (M.U.) per milligram of protein. All data were graphed and analysed using GraphPad Prism software, version 10.0 (www.graphpad.com).

### FLAG-tagging, pulldown and western blot analysis

A triple FLAG-tag was inserted to the N-terminus of uPPE2 in the *ppe2’-lacZ* reporter construct containing the AUGA overlap or the N-terminus of the uPPE2_nostop_ construct in which the AUGA overlap is eliminated using Q5 site-directed mutagenesis (New England Biolabs). The constructs were transformed into *M. smegmatis* wildtype in parallel with *ppe2’-lacZ* or uPPE2_nostop_ constructs without FLAG tags and 60 ml of log-phase (OD_600_ ∼0.6) cultures were prepared for western blot analysis. Cells were pelleted, resuspended and washed three times in 1 ml PBS (pH 7.9), and finally resuspended in 1 ml B-PER™ Bacterial protein extraction reagent (Thermo Fisher Scientific) before disrupting with lysing matrix B in a FastPrep machine (settings: speed = 6.5, time = 40 s, three times with cooling intervals on ice). Pulldowns were carried out with Pierce™ Anti-FLAG Magnetic agarose (Thermo Fisher Scientific) largely according to manufacturer's instructions. Briefly, extracts were cleared by centrifugation and 500 μl of the supernatant was added to 50 μl of the equilibrated beads and incubated at RT for 1 h. Extracts were washed twice with PBS containing 200 mM NaCl, eluted in 100 μl 0.1 M glycine, pH 2.8 and neutralised with Tris pH 8.5. SDS loading buffer was added to all extracts before boiling and loading onto a 4–20% Mini-PROTEAN Tris-glycine gradient gel (Bio-Rad). Proteins were separated by SDS-PAGE and transferred to a PVDF membrane. The FLAG-tagged protein was detected by incubating the milk powder-blocked membrane with mouse monoclonal anti-FLAG primary antibodies (Sigma-Aldrich) diluted at 1:1000, followed by incubation with peroxidase-conjugated polyclonal goat anti-mouse IgG (Jackson ImmunoResearch) diluted at 1:1000. β-galactosidase was detected with anti-LacZ antibodies (Thermo Fisher) diluted at 1:1000, followed by incubation with peroxidase-conjugated polyclonal goat anti-rabbit IgG (Jackson ImmunoResearch) diluted at 1:1000. Signals were visualized on a Li-Cor Odyssey Fc Imager (Licor).

### In-line probing

In-line probing was performed as described by Regulski and Breaker (62). Riboswitch RNA was transcribed using the Megascript T7 High Yield Transcription Kit (Invitrogen) from amplicons generated by PCR using the primers listed in Supplementary Table S1. Transcribed RNA was extracted from denaturing 8% polyacrylamide gel in 500 μl crush-and-soak buffer (0.5 mM sodium acetate pH 5.2, 0.1% SDS, 1 mM EDTA pH 8.0, 30 μl acid phenol:chloroform) and precipitated in ethanol. The yield and purity of the transcribed product were analysed on a Nanodrop 2000. Dephosphorylation was done using calf intestinal alkaline phosphatase (1 U/μl) (Life Technologies). Each 25-μl 5′ end-labelling reaction contained 10 pmol RNA, 133 nM ^32^P γ-ATP (6000 Ci/mmol; Hartmann Analytic GmbH), 1 μM unlabelled ATP, and 25U T4 polynucleotide kinase (10 U/μl) (New England Biolabs). The radiolabelled RNA was purified by PAGE and resuspended in 40 μl nuclease-free water. For in-line probing, reactions contained 2 μl radiolabelled RNA (∼25 nM), 1 × in-line reaction buffer (50 mM Tris–HCl pH 8.3, 20 mM MgCl_2_, 100 mM KCl), and the desired concentration of AdoB_12_, MeB_12_, HyB_12_ or CNB_12_ in 20-μl total volume. Reactions were incubated on a heat block maintained at 30 ºC for 20 h and quenched with an equivalent volume of 2 × colourless gel-loading solution (10 M urea, 1.5 mM EDTA pH 8.0). In-line reaction products were separated by denaturing 6–10% polyacrylamide gel electrophoresis (PAGE) at 45W. The gels were dried and exposed to radiosensitive screens and the data were collected on a Typhoon FLA 9500 (GE Healthcare). The dissociation constants (*K*_d_) of the riboswitches were calculated from the in-line probing data in Figures 3A and 5A, by plotting the fraction of RNA cleaved at ligand-sensitive (cleaved) sites against the logarithm of AdoB_12_ concentration, using the formula described in (62). In Graphpad prism (version 10.0), the data were fitted using the Richards equation to obtain *K*_d_ values (*metE* riboswitch: *K*_d_ = 20.6 ± 7.2 μM; *ppe2* riboswitch: *K*_d_ = 446 ± 46.0 μM.

### Sequence alignments

The DNA sequences of *M. tuberculosis metE* and *ppe2* leaders stretching from ∼40 nt upstream of the TSS to the first codon of the downstream annotated ORF were used as the input query for nucleotide alignment on the NCBI BLAST tool (63). Matches of >95% identity in representative mycobacteria were extracted and their TSS located by examining their respective -10 elements. The 5′ ends of the shortlisted sequences were trimmed to only –5 nt relative to the TSS. The DNA sequence was converted to RNA prior to alignment using t-coffee with default settings (64). Amino acid sequences of *uPPE2* were similarly aligned using t-coffee default settings for protein alignment (64). The resulting clustalw format alignment files were downloaded and edited using the desktop version of Jalview (version 2.11.2.5) (65).

### RNA secondary structure prediction and visualization

Target RNA sequences and matching folding constraints were loaded on the RNAstructure web servers (66), and the structure prediction software ran using default RNAstructure tools (version 6.4). A MaxExpect file containing a CT-formatted structure was downloaded and converted to a dot-bracket-formatted file, which was used to render the 2D secondary structure using the web-based RNA2drawer app (67).

## Results

### Premature transcription termination in metE and ppe2 leaders

**Figure 2.**
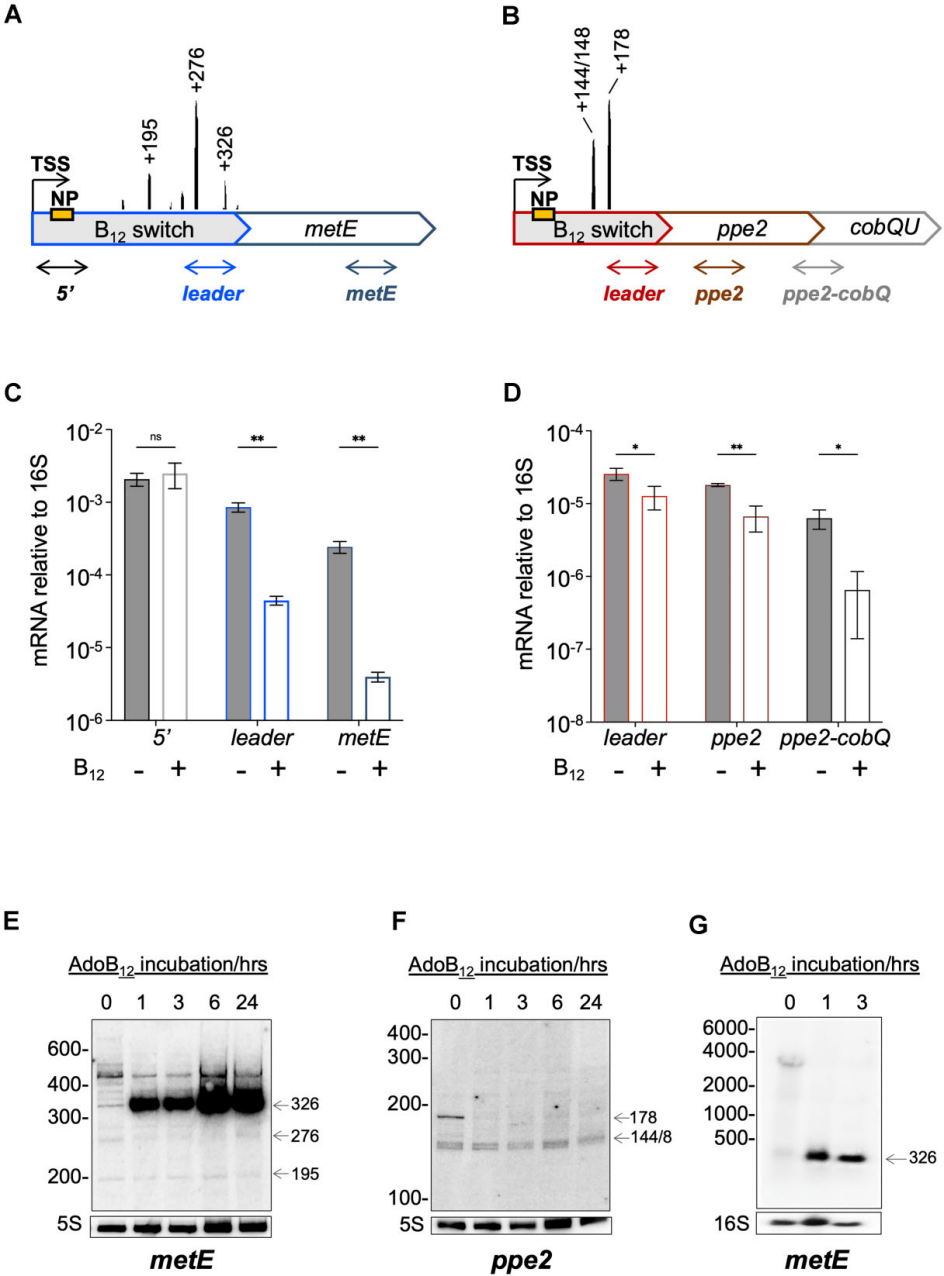
B_12_-dependent changes in *metE* and *ppe2* transcripts. (**A**, **B**) Schematic of *metE* and *ppe2* operons. The full-length of *metE* and *ppe2* leaders are 365 and 292 nt, respectively; ORF lengths (not drawn to scale) are *metE*: 2280 nt, *ppe2*: 1671 nt, *cobQ*: 1485, *cobU*: 525 nt. The precise locations of dominant transcription termination sites (TTS) and their relative coverage according to (13) are indicated. Approximate locations of qRT-PCR amplicons for *metE*/*ppe2* leaders, coding regions and *ppe2-cobQ* junction are indicated as double-ended arrows; exact locations are listed in Materials and Methods. The *metE* 5′ amplicon covers a region upstream of the observed dominant TTS peaks. (**C**, **D**) qRT-PCR of amplicons indicated in (A) and (B) before and 1 h post AdoB_12_ addition; note different scales. Data represents mean ± standard deviation of at least three biological replicates, *P*-values *t*-test: * *P*< 0.05; ** *P*< 0.01. (E, F) Northern blots (vertical, 8% acrylamide) showing changes in short transcripts derived from *metE* (**E**) and *ppe2* (**F**) leaders before and after AdoB_12_ incubation with probes hybridizing to 5′ end of mRNA (orange boxes labelled NP in A and B, above). Arrows right of blots indicate signals corresponding to dominant TTS peaks in A and B, above. G. Horizontal, 1% agarose northern blot using the same *metE* probe as in (E). Arrow indicates a 326-nt transcript corresponding to the dominant, terminated signal in (E).

We recently mapped multiple premature transcription termination sites (TTS) associated with RNA leaders including those of *metE* and *ppe2* in *M. tuberculosis* cultures grown in standard conditions (13). The TTS patterns within these two leaders indicated multiple TTS thoughout the *metE* leader, compared to two, closely spaced TTS in the *ppe2* leader, suggesting differences in the regulation of the switches (Figure 2A and B) (13). To explore how B_12_ might affect growth and *metE* and *ppe2* transcription, we grew cultures of *M. tuberculosis* to OD_600_ ∼0.6 before adding 10 μg/ml AdoB_12_; notably, this did not affect the growth rate (Supplementary Figure S1A). RNA was isolated before and 1 hour after the addition of AdoB_12_, and analysed by quantitative RT-PCR (qRT-PCR) targeting the leader and the coding regions of *metE* and *ppe2* (Figure 2A and B). Using qRT-PCR primers targeting the *metE* TSS-proximal region, we observed no significant change in the RNA levels upon the addition of AdoB_12_ (5′ amplicon, Figure 2C). However, the addition of AdoB_12_ had a profound effect further downstream with a 19-fold decrease in the level of leader amplicon and a 60-fold decrease in *metE* amplicon, reflecting a reduction in transcript levels (Figure 2C). Combined with the mapped TTS, these data suggest that AdoB_12_ induces transcription termination at multiple sites in the *metE* mRNA. By comparison, the changes in *ppe2* transcript levels were more modest, with leader RNA decreasing ∼2-fold and *ppe2* RNA reducing ∼2.5-fold following AdoB_12_ addition (Figure 2D).

The *cobQU* genes downstream of *ppe2* are associated with cobalamin synthesis (31,49), making them likely targets of B_12_-dependent control. Therefore, to determine if *cobQ* is co-transcribed with *ppe2* and thus regulated by the B_12_ switch, we performed qRT-PCR across the *ppe2-cobQ* junction. The results indicated that *ppe2* and *cobQ* are co-transcribed and that RNA levels decrease 10-fold after AdoB_12_ addition, suggesting that *cobQ* is also regulated by the riboswitch (Figure 2D). RT-PCR did not amplify across the *cobQ-cobU* junction, suggesting that either *cobU* is not part of the operon or its expression was below the detection limit, which we consider more plausible (Supplementary Figure S1B). In summary, the results suggest transcriptional polarity in both *loci*, albeit to a lesser degree in *ppe2* than in *metE*.

To confirm the notion of premature termination, we performed northern blotting of RNA at times 0, 1, 3, 6 and 24 hours post AdoB_12_-addition using a probe that hybridized to the 5′ end of each leader. The transcript pattern before AdoB_12_-addition reflected the TTS mapping with multiple signals for *metE* and only a few for *ppe2*; moreover, some of the signals on the blots corresponded to the dominant TTS signals indicated in panels A and B (indicated by arrows in Figure 2E and F). The addition of AdoB_12_ led to one primary but opposite B_12_-dependent change within each leader: a signal corresponding approximately to the TTS at +326 nucleotide (nt) position within the *metE* leader increased substantially upon AdoB_12_ addition, suggesting premature termination of transcription (Figure 2E), while a signal assumed to correspond to the +178-nt TTS within the *ppe2* leader disappeared (Figure 2F). To validate that the *metE* signal around +326 nt was due to premature termination of transcription, we repeated the northern blot with horizontal agarose gels, allowing for detection of larger transcripts including the full-length (∼3 kb) mRNA using the same probe. The image in Figure 2G indicates the presence of a ∼3 kb transcript (at time 0), which upon the addition of AdoB_12_ is replaced by a transcript <500 nt in agreement with the 326-nt transcript observed in Figure 2E. Together, these results strongly support the notion of B_12_-dependent termination of transcription within the *metE* leader. Nevertheless, we cannot rule out that the actual termination site is located downstream of +326 nt, in which case the 3′ end is a result of post-termination trimming. The *ppe2* results do not suggest extensive premature termination of transcription, which corroborates substantial differences between the two riboswitches.

### The metE and ppe2 aptamers display variable selectivity for B_12_ isoforms

To ascertain direct interactions between B_12_ and the *metE* and *ppe2* aptamers and to determine the ligand binding properties of each, we generated transcripts for in-line probing analysis (62) by *in vitro* transcription. Both transcripts covered the region between the TSS and a few bases downstream of their respective most distal leader TTS. Thus, the size of the *metE* riboswitch transcript was 345 nt, while the *ppe2* transcript was 191 nt. First, we analysed the interactions between the two riboswitches and four common B_12_ isoforms: AdoB_12_, MeB_12_, HyB_12_ and CNB_12_. In-line probing reactions contained at least a 4-log excess concentration of ligand (1 mM) over that of RNA. In the case of *metE*, AdoB_12_ induced the strongest modulation signals, while MeB_12_, HyB_12_ or CNB_12_ resulted in little to no change (Supplementary Figure S2). In contrast, all four B_12_ isoforms resulted in similar cleavage patterns and signal intensities in the *ppe2* transcript with the possible exception of C48–C50, where only AdoB_12_ caused a reduction in the cleavage signal (Supplementary Figure S2). In summary, our results indicate that the regions flanked by the TSS and the distal TTS are sufficient for ligand binding in both riboswitches and while the *metE* riboswitch is apparently selective for AdoB_12_, the *ppe2* riboswitch seems able to accommodate all four B_12_ isoforms equally well.

### B_12_-binding leads to occlusion of the metE translation initiation region

**Figure 3.**
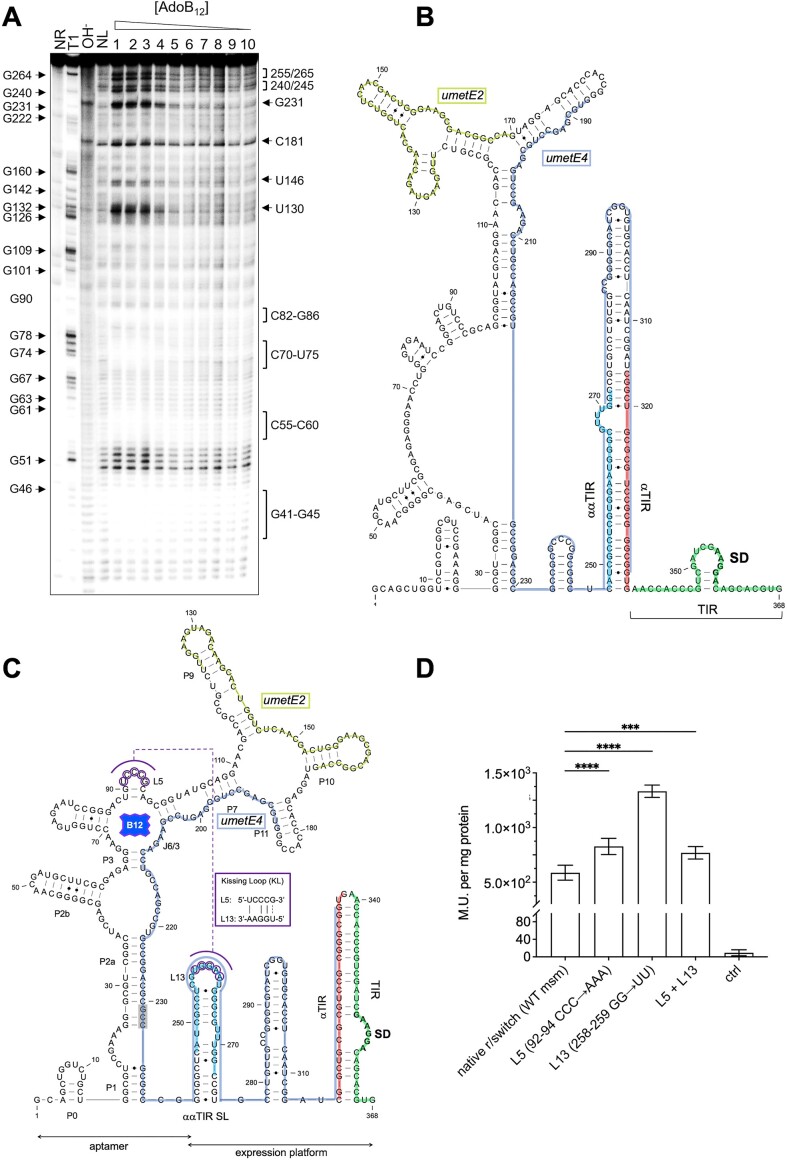
In-line probing of the *metE* riboswitch. (**A**) Cleavage pattern of the *metE* riboswitch RNA over a concentration gradient of AdoB_12_ (lanes, 1: 2 mM; 2: 1 mM; 3: 0.5 mM; 4: 0.1 mM; 6: 10 μM; 6: 1 μM; 7: 0.1 μM; 8: 10 nM; 9: 1 nM; 10: 0.1 nM. T1 – RNase T1; OH^−^ – alkaline digest; NL – no-ligand control; NR – no-reaction. Strongly modified positions are indicated on the right side of the gel (cropped where there was no difference ± ligand). T1-derived G positions indicated on the left. (**B**) Predicted structure of the *metE* switch without ligand. The translation initiation region (TIR) including the Shine-Dalgarno (SD) sequence is highlighted in green, while the αTIR and ααTIR sequences are highlighted in red and blue, respectively. uMetE2 and uMetE4 are described in the text. (**C**) Predicted structure of AdoB_12_-bound *metE* switch. Paired regions in the aptamer are labelled sequentially (P0–P11). The conserved ‘B_12_ box’ (G199–C215) matches the consensus for this element (68). Bases of L5 that could potentially form a kissing loop (KL) with L13 are highlighted in purple and the base pairs between the loops are shown in the inset. The region of hypercleavage at G231–C233 is highlighted in grey. Other coloured features are the same as in panel B. SL – stem-loop. (**D**) Beta-galactosidase assays of reporters expressed in *M. smegmatis* wildtype strains expressing translational LacZ reporters fused to the native or mutant riboswitch bearing nucleotide substitutions in L5 (92–94AAA→CCC), complementary substitutions in L13 (258–259GG→UU), or both. Data represents mean ± standard deviation of at least three biological replicates. *P*-values one-way ANOVA: *** *P*< 0.001; **** *P*< 0.0001.

To interrogate the ligand sensitivity of the *metE* riboswitch, we performed in-line probing using a range of AdoB_12_ concentrations from 0.1 to 2 mM (Figure 3A), which suggested an approximate dissociation constant (*K*_d_) = 20.6 ± 7.2 μM (Supplementary Figure S3). The in-line probing data were used to apply constraints to a predicted structure of the ligand-bound switch on the RNAstructure web server (66). The resulting structure indicated that the aptamer domain of the *metE* riboswitch is contained within the first 220 nt of the leader largely in agreement with the prediction in Rfam (68). Hence, the region downstream of this position was considered part of the expression platform. Several regions of hypercleavage, including G240/C245 & C255/G265, occur in the proposed expression platform in the presence of AdoB_12_ (Figure 3A). These positions are paired in the predicted unbound structure (Apo-form) but adopt single-stranded conformations in the ligand-bound state (Figure 3B and C). An additional structure not reported in Rfam (68) was predicted at positions 1–14, comprising a short hairpin (P0) that is highly conserved in mycobacteria (Figure 3C; Supplementary Figure S4).

According to the predicted structure, the B_12_ binding pocket in the *metE* switch is enclosed in a four-way junction formed by P3–P6. The conserved ‘B_12_ box’ (26) in this switch stretches from G199 to C215, and is followed immediately downstream by hypercleaved positions at G231–C233 (Figure 3A, C). Issuing from the B_12_ pocket are peripheral elements comprising a bipartite P2 arm and a large P6 extension featuring a second four-way junction formed by P7, P8, P10 and P11 (Figure 3C). Moreover, a stereotypical KL is potentially formed by pseudoknot interactions between three conserved cytidines in L5 and a variable partner loop located in the expression platform. To validate the assignment of the KL and the regions involved, we substituted 92–94CCC in L5 with AAA and 258–259GG in L13 with UU. Separately, both of these mutations led to an increase in expression, potentially indicating loss of regulation. However, expression in the double mutant was restored to near wildtype levels (Figure 3D), confirming our assignment of L5 as part of the KL.

The typical expression platform of B_12_ riboswitches is translational (26), and although there were indications of premature termination of transcription, we found no evidence of an intrinsic terminator within the *metE* switch. Comparing the ligand-bound and Apo-structures in Figure 3B and C we identified a potential expression platform, in which a broad translation initiation region (TIR) including a stretch of 25 nt upstream of the MetE start codon and a likely SD were effectively occluded by a complementary α-TIR stem in the ligand-bound structure (Figure 3C). In the Apo-form, the same α-TIR was fully sequestered by extensively pairing with an αα-TIR, thereby unmasking the TIR (Figure 3B). Finally, the αα-TIR formed a hairpin in the ligand-bound structure, in which the apical loop (C255–U262) presented an ideal pairing partner for L5, thereby linking the KL directly to elements of the expression platform (Figure 3B, C). This structure and the ligand preference for AdoB_12_ suggest that the *metE* switch is a Class I switch.

**Figure 4.**
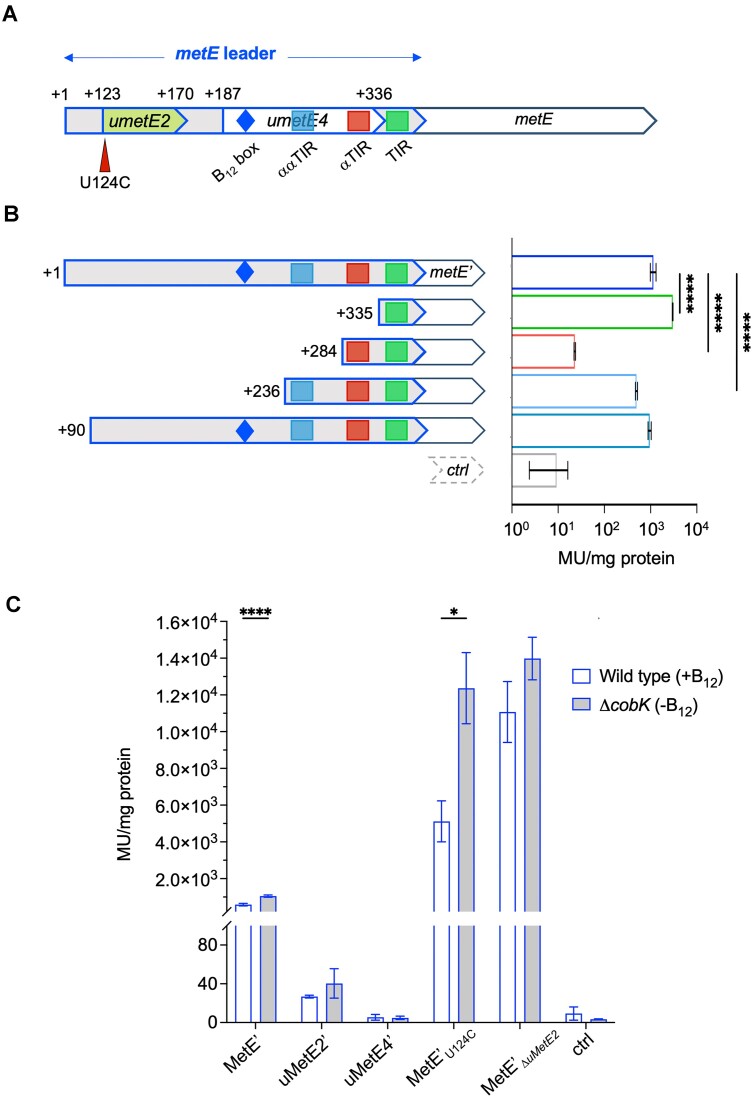
Translation of uMetE modifies MetE expression. (**A**) Outline of the *metE* leader including control elements (TIR, αTIR and ααTIR) identified in Figure 3, the potentially translated leader-encoded peptides, uMetE2 and uMetE4 and location of the U124C mutation. (**B**) Left, outline of reporter constructs for the validation of the translational expression platform using gradual 5′ extensions; numbers indicate 5′ ends of each construct; right, β-gal activities of the reporter fusions shown in Miller Units (MU)/mg protein; note the log-scale. ctrl: no expression control. Data represents mean ± standard deviation of at least three biological replicates. *p*-values One-way ANOVA: **** *P*< 0.0001. (**C**) Assessing the potential for translation of uMetE2 and uMetE4 and the effect of uMetE2 translation. The expression of MetE’-LacZ was measured in the context of wildtype *umetE2*, non-translated *umetE2* (U124C) or Δ*umetE2* (Δ115–188), all expressed in either wildtype *M. smegmatis* (B_12_ producing) or Δ*cobK* (B_12_ deficient). Data represents mean ± standard deviation of at least three biological replicates, *P*-values *t*-test: * *P*< 0.05; **** *P*< 0.0001.

To validate the predicted expression platform and its elements, we made a series of reporter constructs, in which the second codon of *metE* was fused in frame to *lacZ* (*metE’-lacZ*). The upstream edge consisted of gradual 5′ extensions of the *metE* leader to include the predicted TIR, αTIR and the ααTIR, respectively (Figure 4A, B). Predicted secondary structures of these partial leader-constructs are shown in Supplementary Figure S5. To avoid B_12_-dependent folding potentially affecting β-galactosidase (β-gal) activities, all constructs were transformed into a *M. smegmatis* Δ*cobK* mutant incapable of *de novo* B_12_ synthesis (45). The results indicated that the level of MetE’-LacZ expression in the minimal construct (TIR only) was much higher than that of the full-length *metE* leader (∼3-fold increase), suggesting the presence of inhibitory sequences upstream of the basic TIR (Figure 4B). Extending the construct to include the predicted αTIR sequence reduced MetE’-LacZ expression substantially relative to full length leader (>50-fold reduction) (Figure 4B), supporting the notion of translation inhibition via TIR occlusion. A further extension to include the potential ααTIR sequence massively reversed this phenotype yielding only ∼2-fold reduction relative to full length leader (Figure 4B). Put differently, the expression levels in the TIR-only construct were >130-fold higher than when αTIR was included, but only ∼6-fold higher when both αTIR and ααTIR were included (Figure 4B). These data further support the predicted structure-function relationship (Figures 3 and 4B). Finally, an extension to +90 nt restored MetE’-LacZ expression to a similar level as that of the full-length switch (Figure 4B). These findings suggest that the *metE* riboswitch is an ‘OFF’ switch that employs TIR occlusion (i.e. a translational expression platform) via the suggested elements for gene expression control. The formation of the KL directly blocks the proposed ααTIR and reinforce the αTIR-TIR pairing. As previously noted, we also observed a B_12_-enhanced TTS within the *metE* leader associated with a significant reduction in *metE* RNA levels, suggesting that the translational expression platform is augmented by Rho-dependent termination of transcription and/or rapid mRNA degradation following reduced translation.

### A short translated ORF within the metE aptamer suppresses MetE expression

*M. tuberculosis* encodes numerous short, translated ORFs (uORFs) upstream of annotated genes including genes controlled by riboswitches (13,61,69). The *metE* riboswitch encodes four potentially translated uORFs (*umetE1*-*4*) based on credible SD motifs and associated start codons at +88 nt (CUG), +123 nt (UUG), +140 nt (CUG) and +187 nt (GUG) (Figure 3B, C). Ribosome binding and translation within a riboswitch aptamer will likely impact its folding and hence, the function of the switch. To investigate potential functions of *metE* uORFs, we first made in-frame reporter gene fusions to assess their expression. As translation in mycobacteria rarely initiates with CUG start codons (13,61,69,70), we pursued only *umetE*2 (+123 nt to + 170 nt) and the larger *umetE4* (+187 nt to +336 nt) as potentially translated uORFs (Figure 3B, C). The fusions, covering the region upstream of the putative SD motifs to 8 codons of uMetE2 (uMetE2’-LacZ) or 5 codons of uMetE4 (uMetE4’-LacZ), respectively, were expressed in a wildtype (i.e. B_12_ proficient) *M. smegmatis* strain. The resulting β-gal assays indicated low levels of uMetE2’-LacZ expression compared to the much more highly expressed MetE’-LacZ, whereas the expression level of uMetE4’-LacZ was no higher than background (Figure 4C). This suggests uMetE2 may be translated *in vivo* thereby potentially affecting riboswitch function.

To investigate whether translation of uMetE2 had implications for MetE expression and B_12_-sensing, we mutated the start codon of uMetE2 to a non-start codon (UUG⟶UCG) within the context of the full-length MetE’-LacZ fusion (Figure 4B; U124C). The construct was transformed into wildtype and Δ*cobK* (B_12_-deficient) *M. smegmatis* backgrounds to assess if B_12_-sensing was intact in the mutant. To our surprise, this mutation led to a >10-fold increase in MetE’-LacZ expression in both wildtype and Δ*cobK* backgrounds, while the B_12_-dependent change was maintained (Figure 4C). This finding suggested that although the mutation dramatically altered overall expression, it did not impair B_12_ sensing. Conversely, deletion of the entire *umetE2* (segment spanning +115 nt to +188 nt) led to complete loss of B_12_-sensing, while also increasing expression of MetE’-LacZ (Figure 4C). In summary, these results suggest that translation of the aptamer-encoded uMetE2 is likely to contribute to the overall control of MetE expression. At this stage, we are unable to clarify whether this involves the uMetE2 peptide.

### ppe2 is preceded by a riboswitch-controlled translated leader

**Figure 5.**
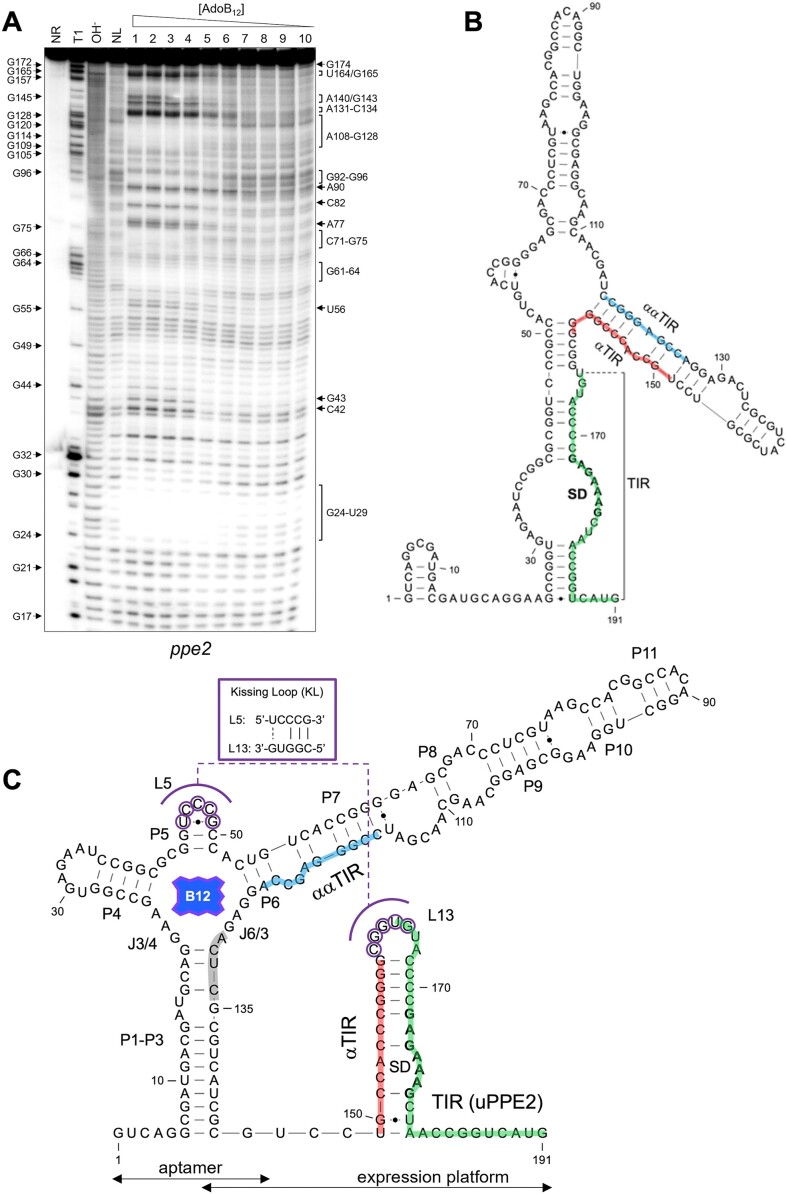
Structure of *ppe2* riboswitch. (**A**) The cleavage pattern of the *ppe2* switch over a concentration gradient of AdoB_12_. Strongly modulated positions are indicated by labels on the right side of the gel. G positions are shown on the left. NR – no-reaction control; T1 – RNase T1 ladder; OH^−^ – alkaline digest ladder; NL – no-ligand control; lanes, 1: 4 mM; 2: 2 mM; 3: 1 mM; 4: 0.5 mM; 5: 0.1 mM; 6: 10 μM; 7: 1 μM; 8: 100 nM; 9: 10 nM; 10: 1 nM. (**B**) Predicted secondary structure of the Apo-form of the *ppe2* switch. The translation initiation region (TIR) including the SD motif is highlighted in green, while the αTIR and ααTIR sequences are highlighted in red and blue, respectively. (**C**) AdoB_12_-bound *ppe2* switch. Paired regions in the aptamer are labelled sequentially from P1 to P11. The ‘B_12_ box’ located G121–G135 matches the consensus (68). The region of hypercleavage at A131-C134 is highlighted in grey. Bases of L5 that could potentially form a kissing loop (KL) with L13 are highlighted in purple and the base pairs between them are shown in the inset.

The results presented so far suggested multiple functional differences between the *metE* and *ppe2* switches. Therefore, to investigate in more detail the structure-function relationship of the *ppe2* switch, we performed in-line probing of the *ppe2* leader (from +1 nt to +191 nt) using a range of AdoB_12_ concentrations from 1 to 4 mM (Figure 5A). The results indicate that cleavage of the *ppe2* riboswitch was modulated by AdoB_12_ in a dose-dependent manner, with strongly protected regions at G24-U29, G61-G64 and A108-G128 and sections of hypercleavage at A131-C134 and U164/G165 (Figure 5A). This in turn suggests that the sequences required for ligand binding, i.e. the aptamer, are contained within the probed fragment. However, the affinity towards AdoB_12_ was found to be considerably lower than that of the *metE* aptamer (*ppe2* switch *K*_d_ = 446.1 ± 46.0 μM versus *metE* switch *K*_d_ = 20.6 ± 7.2 μM) (Supplementary Figure S3). We predicted the secondary structure of the ligand-bound *ppe2* riboswitch using probing-derived folding constraints and a previously proposed KL interaction between L5 and L13 for this switch (26) (Figure 5C). Similar to that of the *metE* riboswitch, the B_12_ binding pocket of the *ppe2* riboswitch was also enclosed in a four-way junction formed by the paired segments P3–P6. However, unlike the *metE* switch, the *ppe2* riboswitch contained a fused P1–P3 arm and a truncated P6 extension (Figure 5C). The predicted structure suggests that the AdoB_12_-induced masking of positions A108–G128 are likely due to a combination of base pairing and ligand contacts (Figure 5A, B). Moreover, this structure mirrors that of the *metE* switch where strong cleavage signals are observed immediately downstream of the B_12_ box (Figure 5A; positions A131–C134).

The region immediately upstream of the annotated *ppe2* ORF is devoid of any obvious SD motifs, but a potentially translated (13) and partially conserved uORF (uPPE2) located between + 189 nt and the start of *ppe2* appears to have a SD motif (Figure 5 and Supplementary Figure S6). In the predicted ligand-bound state, the uPPE2 TIR including this SD motif is partially masked by an αTIR stem, and together these elements form a hairpin that harbours a likely L5 pairing partner (L13, Figure 5C).

In the predicted Apo-structure, the αTIR is sequestered by a purine-rich ααTIR sequence, while the SD motif and its downstream region are rendered more accessible for ribosome binding (Figure 5B). Notably, the ααTIR precedes and partially overlaps the B_12_ box (Figure 5C). In summary, this structures suggests that the probed 191-nt fragment contains both aptamer and expression platform, and we conclude that the *ppe2* switch presents as a very compact, translational ‘OFF’ switch, potentially controlling the expression of uPPE2 in addition to *ppe2* and *cobQU*.

**Figure 6.**
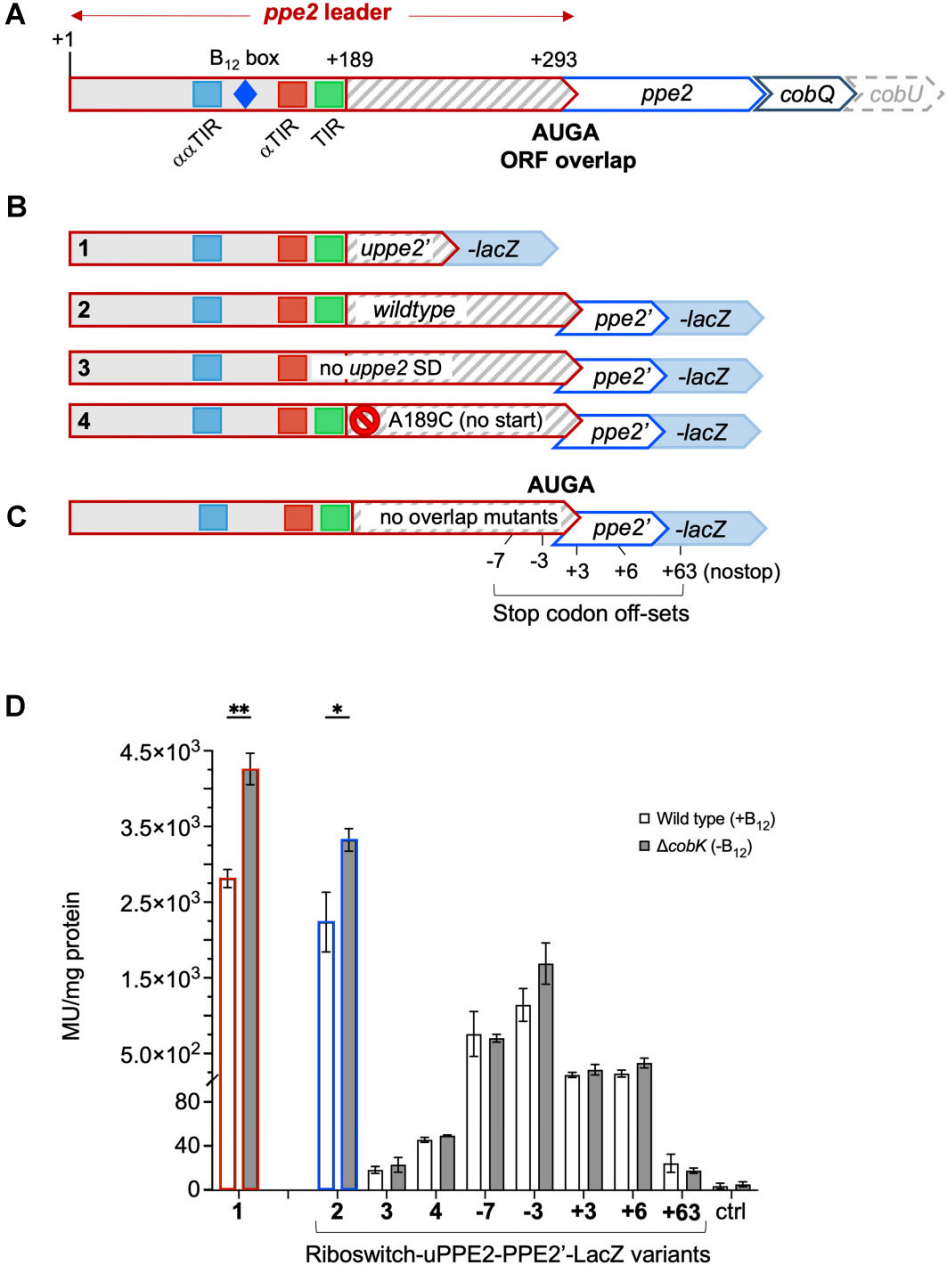
PPE2 are uPPE2 are co-regulated and co-translated. (**A**) Outline of the *ppe2* operon indicating the regulatory elements, the out-frame uPPE2 ORF and the AUGA overlap. (**B**) Outline of *uppe2’-lacZ* and *ppe2’-lacZ* reporter fusions including non-translated uPPE2 variants (no SD and no start (A189C)); note that all constructs have the full leader, but 1 is the only *uppe2’-lacZ* fusion, while the remaining are all *uppe2*-*ppe2’-lacZ* fusions; 2: *uppe2-uppe2’-lacZ*, 3*: uppe2_noSD_-uppe2’-lacZ, 4: uppe2_A189C_-uppe2’-lacZ*. (**C**) Locations of the artificially introduced early or late uPPE2 stop codons −3, −7, +3 and +6 indicate the codon distance between wildtype and new UGA stop codons, +63 (nostop), i.e. native stop codon eliminated, while next natural stop codon is located 63 codons downstream. (**D**) Result of β-gal reporter assays showing riboswitch mediated regulation of expression of uPPE2’-LacZ and PPE2’-LacZ, and uPPE2-dependent translation of PPE2 in wildtype and Δ*cobK* backgrounds (activity is reported as Miller units (MU)/mg total protein). Numbers 1–4 correspond to constructs shown above in (B) and (C). −3 to + 63 indicate the codon distance between wildtype and new UGA stop codon. Data represent mean ± standard deviation of three biological replicates. ctrl – no expression control, *P*-values *t*-test: * *P*< 0.05; ** *P*< 0.01.

To assess whether uPPE2 is the first regulatory target in the *ppe2* operon, we made separate in-frame LacZ fusions of PPE2 and uPPE2, which included the upstream riboswitch and 3 codons of uPPE2 for the *uPPE2’-lacZ* construct and 13 codons of PPE2 for *PPE2’-lacZ*, to incorporate the PPE motif located at residues 10–12 of PPE2 (Figure 6A, B). The constructs were transformed into *M. smegmatis* wildtype and Δ*cobK* and subsequent β-gal assays indicated that uPPE2’-LacZ expression was higher than that of PPE2’-LacZ (Figure 6D). The partial conservation together with the high level of expression suggested that uPPE2 was functional and the first target in the *ppe2* operon.

### Expression of PPE2 depends on translation of the upstream ORF

Hundreds of adjacent ORFs in *M. tuberculosis* have been found to share a 4-nt N*UGA* overlap between stop and start codons, which may affect translation of the downstream ORF (13,59). As mentioned above, the annotated PPE2 ORF lacks a SD motif, but it shares an A*UGA* stop/start overlap with the out-of-frame uPPE2 uORF (Figure 6A). We therefore hypothesised that translation of PPE2 might depend on translation of this uORF. To ascertain if this were the case, we introduced mutations that prevented translation initiation of uPPE2 in the fully-leadered PPE2’-LacZ fusion. In one construct, we eliminated the uPPE2 SD motif by replacing the purines with their complementary pyrimidines (uPPE2_noSD_); in the other, we changed the uPPE2 AUG start codon to the non-start CUG (uPPE2_A189C_) (Figure 6B). Both uPPE2_noSD_ and uPPE2_A189C_ mutants exhibited significantly severely diminished PPE2’-LacZ expression (Figure 6D), suggesting that expression of PPE2 is strictly dependent on uORF translation. This may be due to Rho-dependent termination of transcription, as rho-binding sites become exposed in the absence of uPPE2 translation. Alternatively, it could be the result of translational coupling between the uPPE2 and PPE2 ORFs or a combination of the two.

To dissect the mechanism underlying this translational dependence, we first eliminated the native stop codon in the uPPE2-PPE2’-LacZ reporter construct, such that the uORF was extended with 63 codons until the next stop, located in *lacZ* (uPPE2_(nostop)_-PPE2’-LacZ). We subsequently introduced new stop codons 3 and 7 codons upstream (early stop) or 3 and 6 codons downstream (late stop) of the PPE2 start codon (Figure 6C). All constructs were expressed in *M. smegmatis* (wildtype and Δ*cobK*) and β-gal activity determined. The results indicated that early/premature stops led to a gradual reduction in PPE2’-LacZ expression and B_12_-sensing (Figure 6D). Conversely, introducing the new stop codons downstream of the PPE2 start codon led to a more dramatic reduction in PPE2’-LacZ expression and B_12_ sensing with the uPPE2_(nostop)_ mutant exhibiting activity in the same range as the uPPE2_noSD_ and uPPE2_A189C_ mutants (Figure 6D). This suggests that Rho-dependent termination did not account for the reduced expression and moreover, that a tight stop/start overlap and a forward movement of the ribosome are necessary for efficient expression of the downstream ORF.

### Translation of PPE2 proceeds primarily via termination-reinitiation

**Figure 7.**
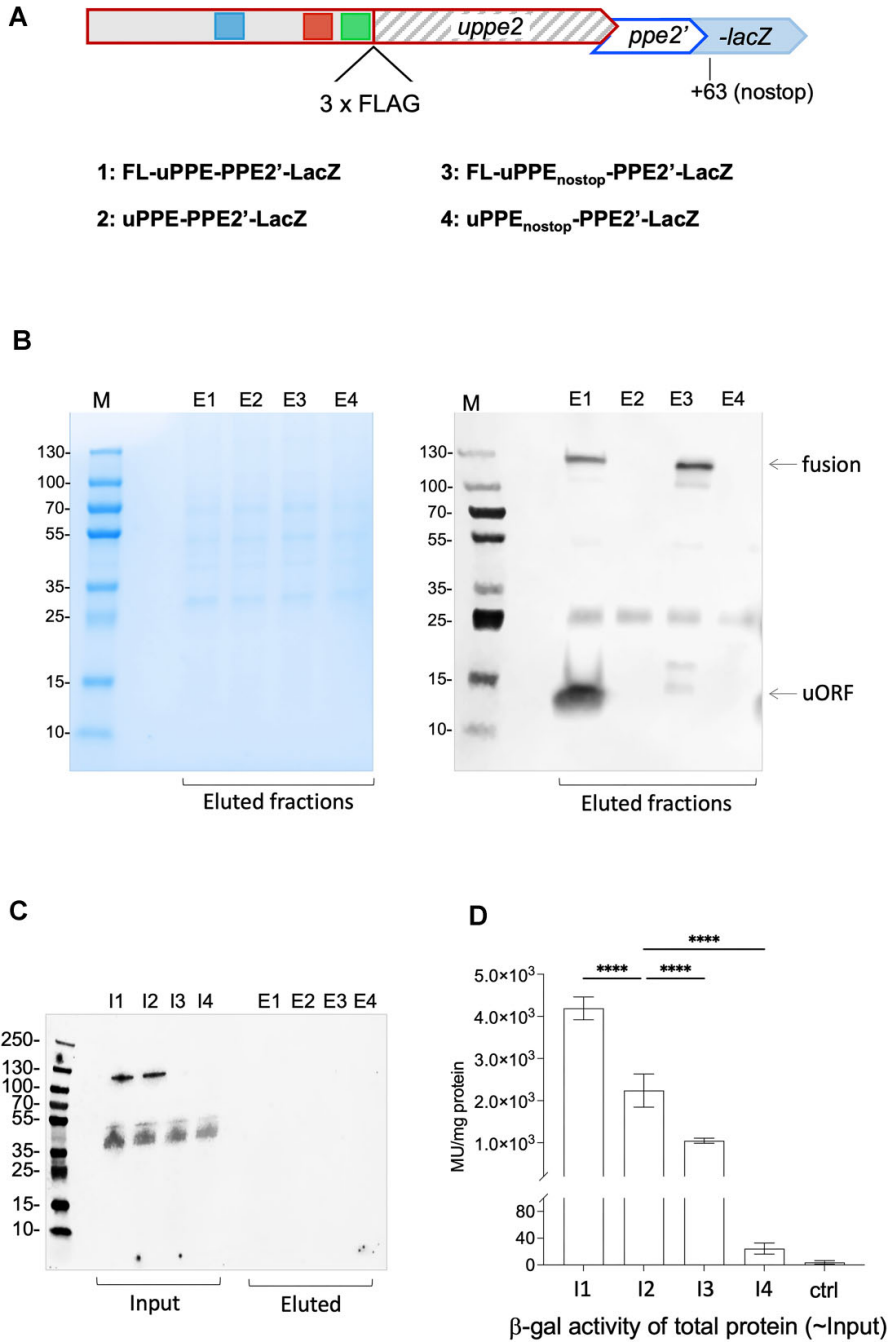
Expression of a uPPE2-PPE2 fusion protein. (**A**) Schematic outlining the FLAG-tagged uPPE2 within the uPPE2-PPE2'-LacZ constructs; numbers 1–4 indicates the nature of each construct (± FLAG, wildtype or nostop) shown in gel (A) Western blots (B and C) and β-gal assays (D). (**B**) Coomassie stained SDS-PAGE (left) and western blot (right) of FLAG-purified (Eluted) cell extracts from *M. smegmatis* expressing FLAG-tagged uPPE2 (lanes E1 & E3), un-tagged uPPE2 (lanes E2 & E4). Arrows indicates likely FLAG-uPPE2 and 120-kDa FLAG-tagged uPPE2-PPE2’-LacZ fusion protein. M: Page Ruler Plus, protein ladder. (**C**) Anti-LacZ western blot of raw and FLAG-tagged cell extracts from the four LacZ-fusion strains. (**D**) β-gal activities of the same four strains. Data represents mean ± standard deviation of at least three biological replicates. *P*-values one-way ANOVA: **** *P*< 0.0001.

Translational coupling between overlapping ORFs could proceed via a Termination-Reinitiation (TeRe) mechanism leading to production of two separate polypeptides (13,59), or via stop codon suppression combined with a frameshift without peptide release, which should generate a large fusion protein encoded by the two ORFs (71,72). To determine which of these scenarios applied, we added an N-terminal FLAG-tag to uPPE2 in the uPPE2-PPE2’-LacZ reporter fusion (Figure 7A). If TeRe were taking place, we would expect a small, FLAG-tagged uORF of 6.3 kD and an un-tagged PPE2’-LacZ fusion of 114 kD. However, if stop codon suppression were taking place, we would expect a large, FLAG-tagged uPPE2-PPE2’-LacZ fusion of 120 kD. In addition, we included an N-terminal FLAG-tag of the uPPE2_nostop_-PPE2’-LacZ mutant, which should produce a larger (∼13 kDa) FLAG-uORF product, while the fate and nature of PPE2 and LacZ remained unknown.

All constructs were expressed in wildtype *M. smegmatis* and cell extracts from FLAG-tagged and isogenic, non-FLAG-tagged reporter strains were prepared. The cell extracts were first enriched using anti-FLAG pulldowns (see Materials and Methods), and the purified extracts were separated by SDS-PAGE followed by either Coomassie staining or western blotting.

Coomassie staining of gels suggested a high degree of purification in eluates with few, faint signals mostly between 25 and 70 kD compared to input, flowthrough and bead retained fractions (Figure 7B and Supplementary Figure S7). Western blotting using anti-FLAG antibodies indicated the FLAG-specific enrichment of two main signals outside this range >100 kDa and <15 kDa (Figure 7B, lanes E1 and E3). These correspond roughly in size to the FLAG-uPPE2-PPE2’-LacZ fusion and the FLAG-uORF, although the potential FLAG-uORF exhibited aberrant mobility, which we ascribe to its C-terminal poly-proline stretch (Figure 7B, lane E1; Supplementary Figure S7). This suggests that the uORF is mainly expressed as a separate peptide, supporting the notion of a TeRe mechanism. In addition, we observed a slightly fainter signal of ∼120 kDa, corresponding in size to the FLAG-uPPE2-PPE2’-LacZ fusion, suggesting that in a few cases, a frameshift occurred and the fusion was made.

Eliminating the native uORF stop codon (FLAG-uPPE2_nostop_-PPE2’-LacZ) resulted in further reduced mobility of the FLAG-uORF signal, which had an expected size of ∼13 kDa and harboured an additional polyproline stretch compared to the wildtype uORF. In this case, we observed two signals corresponding to ∼18 kD and ∼13 kD, respectively (Figure 7B, lane E3). Given the aberrant mobility of the wildtype uORF and the additional poly-proline stretch in the uPPE2_nostop_ construct, we assume that the ∼18 kD signal corresponds to the tagged uORF, while the 13 kD signal is likely a degradation product. Together, the two signals were substantially fainter than that of the wildtype FLAG-uORF, suggesting that either less was made or more was degraded, or both (Figure 7B, lanes E1 versus E3). A signal corresponding in size to FLAG-uPPE2-PPE2’-LacZ was marginally stronger in the uPPE2_nostop_ construct (Figure 7B, lane E3), suggesting that eliminating the stop codon resulted in similar or perhaps slightly increased amounts of a large, FLAG-tagged fusion protein in line with the reduced amounts of uORF. The β-gal assays (Figure 6D) had indicated very little functional LacZ was made in the uPPE2_nostop_ strain; yet, of the three possible readings frames, only the one encoding *lacZ* was large enough to produce a protein of this size.

To resolve this conundrum, we repeated the western blot on raw and FLAG-purified cell extracts using anti-LacZ antibodies. The result, shown in Figure 7C, indicated that LacZ could only be detected in the raw extracts, while there was no detectable signal in the FLAG-purified fractions. Given that the FLAG-enriched fractions corresponded to approximately five times as much cell extract as the input fractions, we conclude that the vast majority of LacZ produced was un-tagged, i.e. generated from TeRe. This is in agreement with the finding that the FLAG-uORF displayed the strongest signal (Figure 7B), and further supporting the notion that TeRe is the predominant mechanism behind PPE2’-LacZ expression. The uPPE2_nostop_ mutant did not result in a LacZ-specific signal in the unpurified fractions, suggesting that although there seems to be more of a FLAG-tagged fusion of the anticipated size (Figure 7B, lane E3), substantially less LacZ is made overall, which is in agreement with the β-gal activity shown in Figure 6D.

As a final control, we compared the β-gal activities of the fusions with and without the FLAG-tags. The results supported the finding that regardless of the tag, the wildtype AUGA context led to higher levels of LacZ, seen as higher β-gal activity (Figure 7D). However, to our surprise, we found that in both wildtype (AUGA) and uPPE2_nostop_ context, addition of the FLAG-tag significantly enhanced β-gal activity (Figure 7D), suggesting that the tag had a stabilizing effect on either RNA or protein, or both.

In summary, PPE2 expression depends on translation of uPPE2, which acts as a landing pad for the ribosomes. Translation of PPE2 proceeds mainly via a TeRe mechanism, but in a few cases the UGA stop codon is suppressed and a fusion protein is made. Adding a FLAG-tag led to increased β-gal activity, possibly due to the stabilization of RNA, protein or both.

## Discussion

In the current study, we have combined in-line probing, structure prediction, and biochemical and genetic approaches to compare the gene expression control mechanisms by two riboswitches in *M. tuberculosis*. Both switches are B_12_-sensing ‘OFF’ switches, in agreement with previous observations for the *metE* switch and on B_12_ riboswitches in general (21,26,30,44,45,53,54,73,74). Both riboswitches operate via B_12_-dependent masking and unmasking of the SD sequence, and in the case of *metE* this is accompanied by a massive reduction in mRNA levels, which is likely a result of Rho-dependent termination potentially in combination with mRNA degradation (13). The downregulation of *ppe2* mRNA was less pronounced. However, it is worth noting that the *ppe2* Apo-form starts with a stem−loop, which may increase mRNA stability compared to the ligand-bound form, which has several unpaired 5′ nucleotides (60).

Both riboswitches displayed features typical of B_12_ riboswitches such as the B_12_ box and kissing loop (KL), but there were also some unique features in both switches. For example, while the L5 half of the KL was identical in the two riboswitches, the interacting L13 differed slightly, although still providing similar base pairing. Moreover, the *metE* L13 overlapped with the proposed ααTIR, while *ppe2* L13 was flanked by the αTIR and TIR (Figures 3 and 5). Finally, the *metE* aptamer domain and the expression platform presented as two distinct domains, where the ααTIR was located well downstream of the B_12_ box and separated from the αTIR by ∼60 nt (Figure 3). This contrasted with the *ppe2* switch, where the overall size and distance between individual elements were much smaller. The ααTIR of the *ppe2* switch was located earlier in the switch in the P6/P7 extension, *i.e*. upstream of the B_12_ box, indicating a substantial overlap between the aptamer domain and the expression platform. Moreover, the αTIR was located only ∼20 nt further downstream of the ααTIR (Figure 5).

In addition to the proposed structures, we demonstrate the presence of atypical and distinct regulatory uORF-related features that contribute to downstream gene expression in the two operons. uMetE2 is encoded within the P9-P10 extension of the aptamer upstream of the conserved B_12_ box. Mutating the uMetE2 start codon to eliminate translation significantly increased MetE expression, suggesting that uMetE2 is translated *in situ*, leading to suppression of MetE expression. Presumably, translation of uMetE2 and ligand-induced folding are mutually exclusive, but our results suggested that B_12_-sensing remains. Whether the suppression is a *cis* or *trans* effect, resulting from the peptide, remains unknown, but the fact that uMetE2 shows limited conservation (Supplementary Figure S4) suggests that its function may be related to specific lifestyles of some members of the *M. tuberculosis* complex.

Another rather unexpected feature was the indispensable relay linking the B_12_ aptamer and the *ppe2-cobQ(U)* operon via uPPE2, *i.e*. that PPE2 was expressed in a uPPE2-dependent manner. In the majority of cases, this happened via TeRe at the uPPE2-PPE2 AUGA overlap as evidenced by (i) a large amount of a FLAG-uORF protein and a much smaller amount of a tagged fusion protein; (ii) no detectable LacZ in the FLAG-purified fraction using anti-LacZ antibodies; and (iii) significantly reduced β−gal activity when this AUGA overlap is disrupted. The uPPE2_nostop_ mutant (FLAG-uPPE2_nostop_-PPE2’-LacZ) also produced a fusion protein, which again did not give rise to a detectable signal using anti-LacZ antibodies. Scrutiny of the region revealed that the *lacZ*-encoding frame is the only frame that could generate a product of that size. Moreover, the next natural stop codon in the uPPE2_nostop_ mutant (63 codons downstream) was in an AUGA context. While this stop codon led to termination, as judged by the anti-FLAG western blot, it remains to be seen whether re-initiation and/or frameshift-fusion are enabled in this context. The absence of a LacZ-specific signal in this mutant suggests that re-initiation may not be as efficient as seen in the wildtype context. Additionally, if this AUGA does allow for frameshift-fusion, the resulting LacZ protein would be devoid of over 50 N-terminal residues, which are critical for function (75,76). The increased activity of FLAG-tagged constructs versus their un-tagged counterparts is likely due to stabilization of RNA and/or protein.

To our knowledge, this is the first demonstration of stop codon suppression and translational coupling of a gene pair with overlapping stop and start codons in *M. tuberculosis*. We are currently investigating how the sequence context surrounding the AUGA stop-start overlap might influence this mechanism and how the N-terminal extension might influence PPE2 function.

We argue that the *ppe2* switch qualifies as a new exceptional member of Class IIb B_12_ riboswitches based on the following considerations. Firstly, the *ppe2* switch does not conform to Class I since it has no P8-P12 extension. Secondly, even though it has the GGAA junctional motif at J3/4 similar to previously reported Class IIb switches, it lacks a corresponding UCU motif in the opposite J6/3 junction (54). Moreover, the *ppe2* switch is neither unable to bind AdoB_12_, similar to Class IIa switches, nor AdoB_12_ selective, similar to other Class IIb switches. Interestingly, positions C48−G50, which form the L5 of the *ppe2* switch, were protected from cleavage only in the presence of AdoB_12_ (Supplementary Figure S2), implying that in this switch, the KL might be stabilized only by binding AdoB_12_ and not MeB_12_, HyB_12_ or CNB_12_. Further structural analysis, such as crystallization is required to obtain the full and true images of these switches in their ligand-bound and -unbound constellations (53,73,77).

It has been suggested that PE/PPE genes inserted and expanded at different genomic *loci* (48). Therefore, one can speculate that *ppe2* ‘invaded’ the locus of the cobalamin biosynthetic genes *cobQ/U*, which were initially under the regulatory control of an ancestral B_12_-riboswitch thereby giving rise to this extra and unconventional regulatory mechanism. This notion is supported to some extent by similar scenarios in the Mbox-*pe20*-*mgtC* locus and the recently identified PE-containing uORF in the *glyA2* locus (13).

The existence of B_12_ riboswitch classes with varying ligand selectivities within the same cell raises some interesting questions. It is assumed that *M. tuberculosis* relies on host-derived B_12_, but whether this is obtained by the pathogen as AdoB_12_ or MeB_12_ remains unknown. Moreover, nothing is known about potential pathways involved in converting the scavenged B_12_ isoform or precursor to the relevant riboswitch ligand or cofactor type. Recent evidence suggests that host B_12_ acted as a key factor in shaping the evolution of human- and animal-adapted *M tuberculosis* lineages, which promotes the notion of B_12_ involvement in virulence and host-pathogen cross-talk (40). Therefore, understanding when and how *M. tuberculosis* utilizes its multi-layered riboswitch complexity to sense and adapt to a range of host niches will not only shed light on how habitats shape genomes, but also provide a deeper understanding of general pathogen adaptation during the course of infection. These unusual molecular mechanisms are yet another example of how *M. tuberculosis* challenges our understanding of gene expression control.

## Supplementary Material

gkae338_Supplemental_Files

## Data Availability

The data underlying this article are available in the article and in its online supplementary material.

## Notes

Present address: Terry Kipkorir, Department of Infection Biology, The London School of Hygiene and Tropical Medicine, Keppel Street, WC1E 7HT London, UK.

Present address: Alexandre D’Halluin, Institut de Biologie Physico-Chimique, Université de Paris, 75005 Paris, France.
