# Supplementary material for: A novel regulatory interplay between atypical B12 riboswitches and uORF translation in Mycobacterium tuberculosis
Source: Nucleic Acids Res. 2024 May 6;52(13):7876–92. doi: 10.1093/nar/gkae338 (PMC11260477; doi:10.1093/nar/gkae338)
Supplement: gkae338_Supplemental_Files [file gkae338_supplemental_files.zip › Kipkorir_Supplementary Table_FINAL.docx]

A novel regulatory interplay between atypical B_12_ riboswitches and uORF translation in *Mycobacterium tuberculosis*

Terry Kipkorir^a,b^*, Peter Polgar^a^, Declan Barker^a^, Alexandre D’Halluin^a,c^, Zaynah Patel^a^, Kristine B. Arnvig^a^*

^a^Institute for Structural and Molecular Biology, University College London, Gower Street, WC1E 6BT London, United Kingdom

^b^Current address: Department of Infection Biology, The London School of Hygiene and Tropical Medicine, Keppel Street, WC1E 7HT London, United Kingdom.

^c^Current address: Institut de Biologie Physico-Chimique, Université de Paris, 75005 Paris, France

*Corresponding authors: Kristine Arnvig (k.arnvig@ucl.ac.uk), Terry Kipkorir (terry.kipkorir@lshtm.ac.uk)

Supplementary Table 1

| **Strains** | | | |  |
| --- | --- | --- | --- | --- |
| **Strain** | **Notes** | | **Reference(s)** |  |
| *E. coli* DH5α | Chemically competent *E. coli* cells capable of high efficiency transformation | | New England Biolabs |  |
| *M. smegmatis* mc^2^155 | Wild type strain of transformation-efficient *M. smegmatis* | | (1) |  |
| *M. smegmatis* Δ*cobK* | *M. smegmatis* mutant with unmarked deletion of *cobK* (MSMEG_3875) | | (2) |  |
| *M. tuberculosis* H37Rv | Laboratory-adapted strain of *M. tuberculosis* | | (3) |  |
| **Plasmids** | | | | |
| **Name** | **Description** | | **Reference(s)** | |
| pIRaTE2020 | A derivative of pIRaTE containing *Hin*dIII and *Nco*I restriction cloning sites and a PCL1 core promoter driving the expression of a *lacZ* reporter; hygromycin selectable | | (4, 5) | |
| pTKSW-*ppe2* | A derivative of pIRaTE2020 containing the leader and the first 13 codons of *ppe2* fused in-frame to *lacZ* | | This study | |
| pTKSW-*metE* | A derivative of pIRaTE2020 containing the leader and the first 2 codons of *metE* fused in-frame to *lacZ* | | This study | |
| pTKSW-*uPPE2* | A derivative of pTKSW-*ppe2* containing a site-directed deletion of the region from +198 to +332 of the *ppe2* leader, which generates an in-frame fusion of the leader of *uPPE2* and its first three codons to *lacZ* | | This study | |
| pTKSW-*ppe2_A296U_* | A derivative of pTKSW-*ppe2* containing an *UGA→UGU* mutation in the stop codon of *uPPE2* | | This study | |
| pTKSW-*FLAG-uPPE2* | A derivative of pTKSW-*ppe2* containing 3× FLAG-tag added to the N terminus of uPPE2 (3× FLAG-tag sequence: GATTATAAGGATCATGATGGTGATTATAAGGATCATGATATCGACTACAAAGACGATGACGACAAG) | | This study | |
| pTKSW-*FLAG-uPPE2_nostop_* | A derivative of pTKSW-*ppe2_A296U_* with 3× FLAG-tag added to the N terminus of uPPE2 | | This study | |
| **Oligonucleotides** | | | | |
| **Oligo ID** | **5’ → 3’ sequence** | **Description** | **Reference(s)** | |
| 1.48 | GTCCCATTCCGAACCCGGAAGCTAAGCCTGCCAGCGCCTGTCTC | Northern blot riboprobe targeting the 5S rRNA transcript | (4) | |
| 3.07 | AAGAAGCACCGGCCAACTAC | Forward primer for qRT-PCR targeting the 16S rRNA transcript | (4) | |
| 3.08 | TCGCTCCTCAGCGTCAGTTA | Reverse primer for qRT-PCR targeting the 16S rRNA transcript | (4) | |
| 10.73 | GGCGTCCGAAAGGGCGTCGGCATCGAGCGGGGCAACGATGCCTGTCTC | Northern blot riboprobe targeting the *metE* leader transcript | This study | |
| 10.74 | GGAGCGACCCTCGTAAGCCACGGCCACAGGCTGGAAGGCGCCTGTCTC | Northern blot riboprobe targeting the *ppe2* leader transcript | This study | |
| 11.12 | TAATACGACTCACTATAGGGCAGCTGGTCTGCTGGCGTC | Forward primer to PCR-amplify the template used to transcribe a 345-nt transcript for inline probing of the *metE* switch | This study | |
| 11.11 | GGTGGTTTCACCGCCCGCGGA | Reverse primer to PCR-amplify the template used to transcribe a 345-nt transcript for inline probing of the *metE* switch | This study | |
| 11.09 | CCGGGACTGTAAAGCAGCGGTATG | Forward primer used to generate 92-94CCC>AAA mutation in the pTKSW-*metE* construct via SDM | This study | |
| 11.10 | ATTCTCACCAGGTTCCCTC | Reverse primer used to generate 92-94CCC>AAA mutation in the pTKSW-metE construct via SDM | This study | |
| 11.20 | TAATACGACTCACTATAGGGTCAGGCGATGACGATGCAG | Forward primer to PCR-amplify the template used to transcribe a 191-nt transcript for inline probing of the *ppe2* switch | This study | |
| 12.26 | CATGACCGGTTAGCTTTCT | Reverse primer to PCR-amplify the template used to transcribe a 191-nt transcript for inline probing of the *ppe2* switch | This study | |
| 11.48 | CTAACCGGTCCTGACGCTCCAAA | Forward primer used to generate *uPPE2_A189C_* in the pTKSW-*ppe2* construct via site-directed mutagenesis (SDM) | This study | |
| 11.49 | CTTTCTCGGGGTACACCG | Reverse primer used to generate *uPPE2_A189C_* in the pTKSW-*ppe2* construct via SDM | This study | |
| 11.80 | Phos/AGCTTACTGTCCCGCAGCGGTATGCAGGAACGACCGCCGTCTTGGAAGTAGACAAGCACTGGTCTCC | Used to generate the *uMETE2’-lacZ* construct by oligo annealing and restriction cloning | This study | |
| 11.81 | Phos/CATGGGAGACCAGTGCTTGTCTACTTCCAAGACGGCGGTCGTTCCTGCATACCGCTGCGGGACAGTA | Used to generate the *uMETE2’-lacZ* construct by oligo annealing and restriction cloning | This study | |
| 12.01 | GCCGCCGATGTCCGCCCCGATC | Forward primer used to generate pTKSW-*ppe2_A296U_* via SDM | This study | |
| 12.02 | TGACCGCCGGCGGAGGTG | Reverse primer used to generate pTKSW-*ppe2_A296U_* via SDM | This study | |
| 12.18 | CCATGGATGATCCCGTCG | Forward primer to generate pTKSW-*uPPE2* via SDM | This study | |
| 12.19 | GAGCGTCATGACCGGTTA | Reverse primer to generate pTKSW-*uPPE2* via SDM | This study | |
| 12.20 | Phos/AGCTTAGCGACGGCCAGTAGGAGCACCCACCGGGTGCGAGCCTGCGAGCC | Used to generate the *uMETE4’-lacZ* construct by oligo annealing and restriction cloning | This study | |
| 12.21 | Phos/CATGGGCTCGCAGGCTCGCACCCGGTGGGTGCTCCTACTGGCCGTCGCTA | Used to generate the *uMETE4’-lacZ* construct by oligo annealing and restriction cloning | This study | |
| 16.58 | ACCGCCGTCTCGGAAGTAGAC | Forward primer used to generate *metE_U124C_* in the pTKSW-*metE* construct via SDM | This study | |
| 16.59 | CGTTCCTGCATACCGCTG | Reverse primer used to generate *metE_U124C_* the pTKSW-*metE* construct via SDM | This study | |
| 12.43 | CCATGGATGATCCCGTCGTTTTAATTAG | Forward primer used to generate pTKSW-*uMETE2* via SDM | This study | |
| 12.44 | ACTGGCCGTCGCTTCCCA | Reverse primer used to generate pTKSW-*uMETE2* via SDM | This study | |
| 12.52 | GACGACACCTTGACCGGCGGTCAGCC | Forward primer used to introduce a stop codon at -21 nt relative to *ppe2* start codon in the pTKSW-*ppe2_A296U_* construct via SDM | This study | |
| 12.53 | GCGTCGCGAGCAGCCACG | Reverse primer used to introduce a stop codon at -21 nt relative to *ppe2* start codon in the pTKSW-*ppe2_A296U_* construct via SDM | This study | |
| 12.54 | TGAAGCCGCCGATGTCCGCCC | Forward primer used to introduce a stop codon at -9 nt relative to *ppe2* start codon in the pTKSW-*ppe2_A296U_* construct via SDM | This study | |
| 12.55 | GACCGCCGGCGGAGGTGTC | Reverse primer used to introduce a stop codon at -9 nt relative to *ppe2* start codon in the pTKSW-*ppe2_A296U_* construct via SDM | This study | |
| 12.56 | ATGTCCGCCCTGATCTGGATGGCTTCGC | Forward primer used to introduce a stop codon at +9 nt relative to *ppe2* start codon in the pTKSW-*ppe2_A296U_* construct via SDM | This study | |
| 12.57 | CGGCGGCTGACCGCCGGC | Reverse primer used to introduce a stop codon at +9 nt relative to *ppe2* start codon in the pTKSW-*ppe2_A296U_* construct via SDM | This study | |
| 12.58 | GATCTGGATGACTTCGCCCCCAGAGGTGC | Forward primer used to introduce a stop codon at +18 nt relative to *ppe2* start codon in the pTKSW-*ppe2_A296U_* construct via SDM | This study | |
| 12.59 | GGGGCGGACATCGGCGGC | Reverse primer used to introduce a stop codon at +18 nt relative to *ppe2* start codon in the pTKSW-*ppe2_A296U_* construct via SDM | This study | |
| 12.60 | GTGTACCCCGTCTTTCCTAACCGGTCATGACGCTCCAAACCTTGTC | Forward primer used to generate *uPPE2_flipped SD_* in the pTKSW-*ppe2* construct via SDM | This study | |
| 12.61 | CGCCCCGGGTGGCAGGAC | Reverse primer used to generate *uPPE2_flipped SD_* in the pTKSW-*ppe2* construct via SDM | This study | |
| 14.68 | GCCGGATTTGTATTAGACTAAGCTTGAGTAGGAGATTTTCACCTCCTTTCCTTCCTACCATGGATG ATCCCGTCGTTTTACA | Used to generate a no-expression control via oligo annealing and Gibson assembly of an insert containing purine-pyrimidine substitutions in the *lacZ* SD sequence of pIRaTE2020 | (5) | |
| 14.69 | TGTAAAACGACGGGATCATCCATGGTAGGAAGGAAAGGAGGTGAAAATCTCCTACTCAAGCTTAGTCTAATACAAATCCGGC | Used to generate the no-expression control via oligo annealing and Gibson assembly of an insert containing purine-pyrimidine substitutions in the *lacZ* SD sequence of pIRaTE2020 | (5) | |
| 16.03 | TCGCTGGGTAATCCGCTAAC | Forward primer for qRT-PCR targeting *ppe2* ORF (coding amplicon) | This study | |
| 16.04 | GAATGCGAAGGTTTGCGACA | Reverse primer for qRT-PCR targeting *ppe2* ORF (coding amplicon) | This study | |
| 16.01 | TGGTTCGACACCAACTACCA | Forward primer for qRT-PCR targeting *metE* ORF (coding amplicon) | This study | |
| 16.02 | GCCCTAACGCCTCTTTGAGT | Reverse primer for qRT-PCR targeting *metE* ORF (coding amplicon) | This study | |
| 16.05 | ATCGAGTTGTTGGACATGTTC | Reverse primer for PCR (and qRT-PCR) targeting the *ppe2-cobQ* junction | This study | |
| 16.06 | CGCAGGACTGATCACGTTA | Forward primer for PCR (and qRT-PCR) targeting the *ppe2-cobQ* junction | This study | |
| 16.07 | CTCATCGCCTCGTGGAATGGGC | Forward primer for qRT-PCR targeting the *metE* leader (leader amplicon) | This study | |
| 16.08 | TGCTGTCCTTCGATCGACGGGT | Reverse primer for qRT-PCR targeting the *metE* leader (leader amplicon) | This study | |
| 16.09 | AACCTTGTCTAGCGGTCGGGCC | Forward primer for qRT-PCR targeting the *ppe2* leader (leader amplicon) | This study | |
| 16.10 | CCATCCAGATCGGGGCGGTCAT | Reverse primer for qRT-PCR targeting the *ppe2* leader (leader amplicon) | This study | |
| 16.48 | ACTTGGATGTCGTGTTCGCT | Forward primer for PCR targeting the *cobQ-cobU* junction; used with 16.51 as reverse primer | This study | |
| 16.49 | CTCAGCCAGGCTAGATCGG | Reverse primer for PCR targeting the *cobQ* coding region; used with 16.48 as forward primer | This study | |
| 16.50 | GTGCCATCCCATTCTTCGGG | Forward primer for PCR targeting the *cobU* coding region, used with 16.51 as reverse primer | This study | |
| 16.51 | TGACCAGATGTACCTCATCGC | Reverse primer for PCR targeting the *cobQ-cobU* junction | This study | |
| TK1.89 | ATCGCCTCGTTTAATGGGCGTTTGGC | Forward primer for SDM of *metE* riboswitch 258-58GG to TT; compensatory mutation for 92-94CCC-AAA | This study | |
| TK1.90 | GAGCCGCCGGGCGC | Reverse primer for SDM of *metE* riboswitch 258-58GG to TT; compensatory mutation for 92-94CCC-AAA | This study | |
| TK1.91 | CTGGTCTGCTGGCGTCCGAAAG | Forward primer for qRT-PCR of the 5' end of the *metE* riboswitch (amplifies from +5 to +117) | This study | |
| TK1.92 | CGGTCGTTCCTGCATACCGCTG | Reverse primer for qRT-PCR of the 5' end of the *metE* riboswitch (amplifies from +5 to +117) | This study | |
| TK2.10 | TCAGCCGCCGCTGACCGCCCC | Forward primer used to generate PPE2_no-start_ via SDM | This study | |
| TK2.11 | CCGCCGGCGGAGGTGTCG | Reverse primer used to generate PPE2_no-start_ via SDM | This study | |
| TK2.63 | CATGATATCGACTACAAAGACGATGACGACAAGACGCTCCAAACCTTGTCT | Forward primer used to insert 3× FLAG-tag to N-terminus of uPPE2 via SDM | This study | |
| TK2.64 | ATCCTTATAATCACCATCATGATCCTTATAATCCATGACCGGTTAGCTTTC | Reverse primer used to insert 3× FLAG-tag to N-terminus of uPPE2 via SDM | This study | |
|  | | | | |
| **Gene Blocks** | **5’ → 3’ sequence** | **Description** | **Reference(s)** | |
| gBlock TK-1 | ATTAGACTAAGCTTGCAGCTGGTCTGCTGGCGTCCGAAAGGGCGTCGGCATCGAGCGGGGCAACGATGCTTCGCGAGAGGGAACCTGGTGAGAATCCGGGACTGTCCCGCAGCGGTATGCAGGAACGACCGCCGTCTTGGAAGTAGACAAGCACTGGTCTCAACGACTGGGAAGCGACGGCCAGTAGGAGCACCCACCGGGTGCGAGCCTGCGAGTCCGAAGACCTGCCAGCCGTGCCGGACGCGCCGCGCCCGGCGGCTCATCGCCTCGTGGAATGGGCGTTTGGCCGTGCCTGTTGCCGGGTGCATCGGTGTGCACCTCAATCGGATCGGCTGCGCGTCCGCGGGCGGTGAACCACCCGTCGATCGAAGGACAGCACGTGACCATGGATGATCCCGTCGTTTTA | GeneBlocks fragment containing the full-length *metE* leader and the first two codons of MetE, used to generate pTKSW-*metE* by restriction cloning with *Hin*dIII and *Nco*I restriction sites, underlined. | This study | |
| gBlock TK-2 | ATTAGACTAAGCTTGTCAGGCGATGACGATGCAGGAAGCCGGTGAGAATCCGGCGCGGTCCCGCCACTGTCACCGGGGAGCGACCCTCGTAAGCCACGGCCACAGGCTGGAAGGCGAGGCAAGCAACGATCCGGGAGCCAGGAGACTCGCGTCATCGCGTCCTGCCACCCGGGGCGGTGTACCCCGAGAAAGCTAACCGGTCATGACGCTCCAAACCTTGTCTAGCGGTCGGGCCACCACCACGCTGCTGGGCCTTAACGTGGCTGCTCGCGACGCGACGACACCTCCGCCGGCGGTCAGCCGCCGATGACCGCCCCGATCTGGATGGCTTCGCCCCCAGAGGTGCCCATGGATGATCCCGTCGTTTTA | GeneBlocks fragment containing the full-length *ppe2* leader sequence and the first 13 codons of PPE2, used to generate pTKSW-*ppe2* by restriction cloning with *Hin*dIII and *Nco*I restriction sites, underlined | This study | |
| gBlock TK-3 | CCATTGCCGGATTTGTATTAGACTAAGCTTGCCGGGTGCATCGGTGTGCACCTCAATCGGATCGGCTGCGCGTCCGCGGGCGGTGAACCACCCGTCGATCGAAGGACAGCACGTGACCATGGATGATCCCGTTTTACAACGTC | GeneBlocks fragment containing the partial *metE* leader sequence, used to generate the TIR-αTIR construct by Gibson assembly with *Hin*dIII and *Nco*I restriction sites, underlined | This study | |
| gBlock TK-4 | CCATTGCCGGATTTGTATTAGACTAAGCTTGCCCGGCGGCTCATCGCCTCGTGGAATGGGCGTTTGGCCGTGCCTGTTGCCGGGTGCATCGGTGTGCACCTCAATCGGATCGGCTGCGCGTCCGCGGGCGGTGAACCACCCGTCGATCGAAGGACAGCACGTGACCATGGATGATCCCGTTTTACAACGTC | GeneBlocks fragment containing the partial *metE* leader sequence, used to generate the TIR-αTIR-ααTIR construct by Gibson assembly with *Hin*dIII and *Nco*I restriction sites, underlined | This study | |
| gBlock TK-5 | CCATTGCCGGATTTGTATTAGACTAAGCTTGTCCCGCAGCGGTATGCAGGAACGACCGCCGTCTTGGAAGTAGACAAGCACTGGTCTCAACGACTGGGAAGCGACGGCCAGTAGGAGCACCCACCGGGTGCGAGCCTGCGAGTCCGAAGACCTGCCAGCCGTGCCGGACGCGCCGCGCCCGGCGGCTCATCGCCTCGTGGAATGGGCGTTTGGCCGTGCCTGTTGCCGGGTGCATCGGTGTGCACCTCAATCGGATCGGCTGCGCGTCCGCGGGCGGTGAACCACCCGTCGATCGAAGGACAGCACGTGACCATGGATGATCCCGTTTTACAACGTC | GeneBlocks fragment containing the partial *metE* leader sequence, used to generate the extended TIR-αTIR-ααTIR construct by Gibson assembly with *Hin*dIII and *Nco*I restriction sites, underlined | This study | |

**References**

1. Snapper,S.B., Melton,R.E., Mustafa,S., Kieser,T. and Jr,W.R.J. (1990) Isolation and characterization of efficient plasmid transformation mutants of *Mycobacterium smegmatis*. *Mol. Microbiol.*, **4**, 1911–1919.

2. Kipkorir,T., Mashabela,G.T., Wet,T.J. De and Koch,A. (2021) De Novo Cobalamin Biosynthesis, Transport, and Assimilation and Cobalamin-Mediated Regulation of Methionine Biosynthesis in *Mycobacterium smegmatis*. *J. Bacteriol.*, **203**, e00620-20.

3. Cole,S.T., Brosch,R., Parkhill,J., Garnier,T., Churcher,C., Harris,D., Gordon,S. V, Eiglmeier,K., Gas,S., Barry,C.E., *et al.* (1998) Deciphering the biology of Mycobacterium tuberculosis from the complete genome sequence. *Nature*, **393**, 537–544.

4. Moores,A., Riesco,A.B., Schwenk,S. and Arnvig,K.B. (2017) Expression, maturation and turnover of DrrS, an unusually stable, DosR regulated small RNA in *Mycobacterium tuberculosis*. *PLoS One*, **12**, e0174079.

5. D’Halluin,A., Polgar,P., Kipkorir,T., Patel,Z., Cortes,T. and Arnvig,K.B. (2023) Premature termination of transcription is shaped by Rho and translated uORFS in *Mycobacterium tuberculosis*. *iScience*, **26**, 106465.
