# Supplementary material for: A novel regulatory interplay between atypical B12 riboswitches and uORF translation in Mycobacterium tuberculosis
Source: Nucleic Acids Res. 2024 May 6;52(13):7876–92. doi: 10.1093/nar/gkae338 (PMC11260477; doi:10.1093/nar/gkae338)
Supplement: gkae338_Supplemental_Files [file gkae338_supplemental_files.zip › Kipkorir_suppl_figures.pdf]

Supplementary figures

A

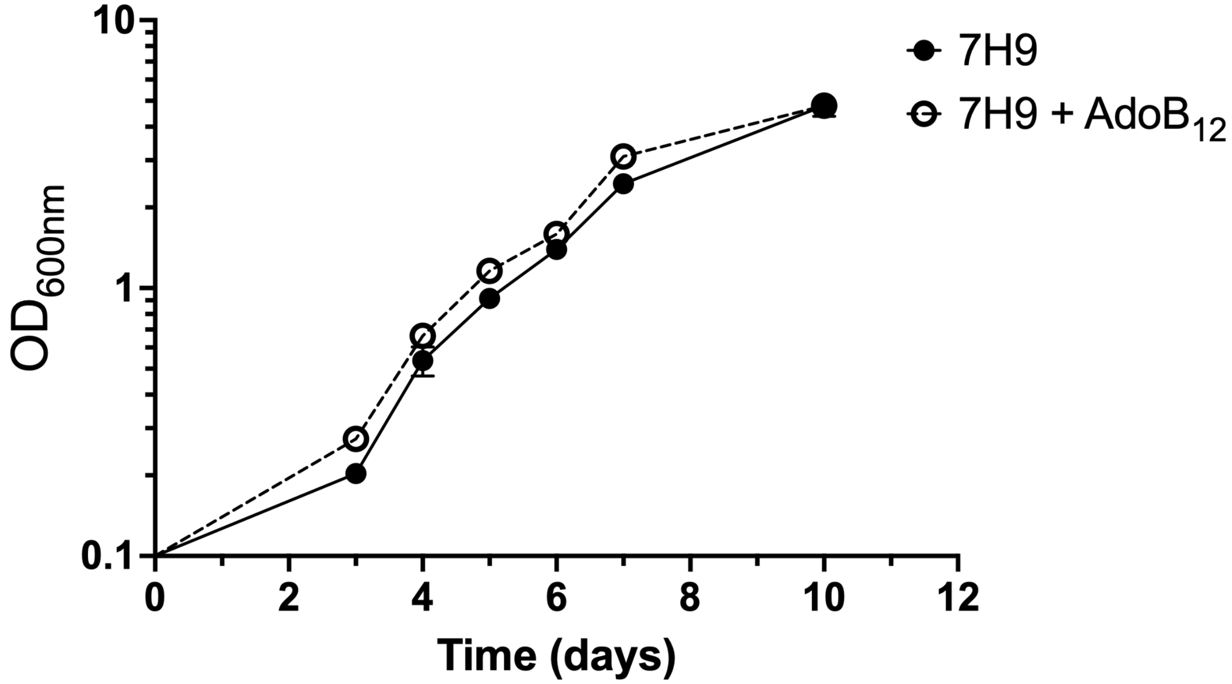

B

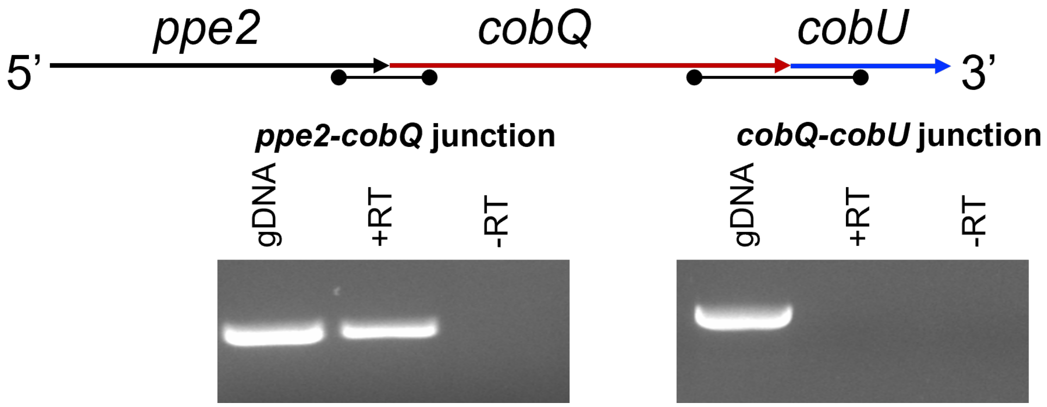

Supplementary Figure 1. **A**. *M. tuberculosis* H37Rv was grown in 7H9/ADC with 0.05% tween-80 and supplemented with or without 10μM $AdoB_{12}$ and OD600nm measurements were taken every 24 h. The data are plotted as mean ± standard deviation of triplicate cultures. **B**. A schematic showing the relative positions of RT-PCR primers (not drawn to scale) targeting the *ppe2-cobQ* junction or *cobQ-cobU* junction. The RT-PCR in **B** was performed using cDNA generated from RNA isolated in *M. tuberculosis* H37Rv cultures without $AdoB_{12}$.

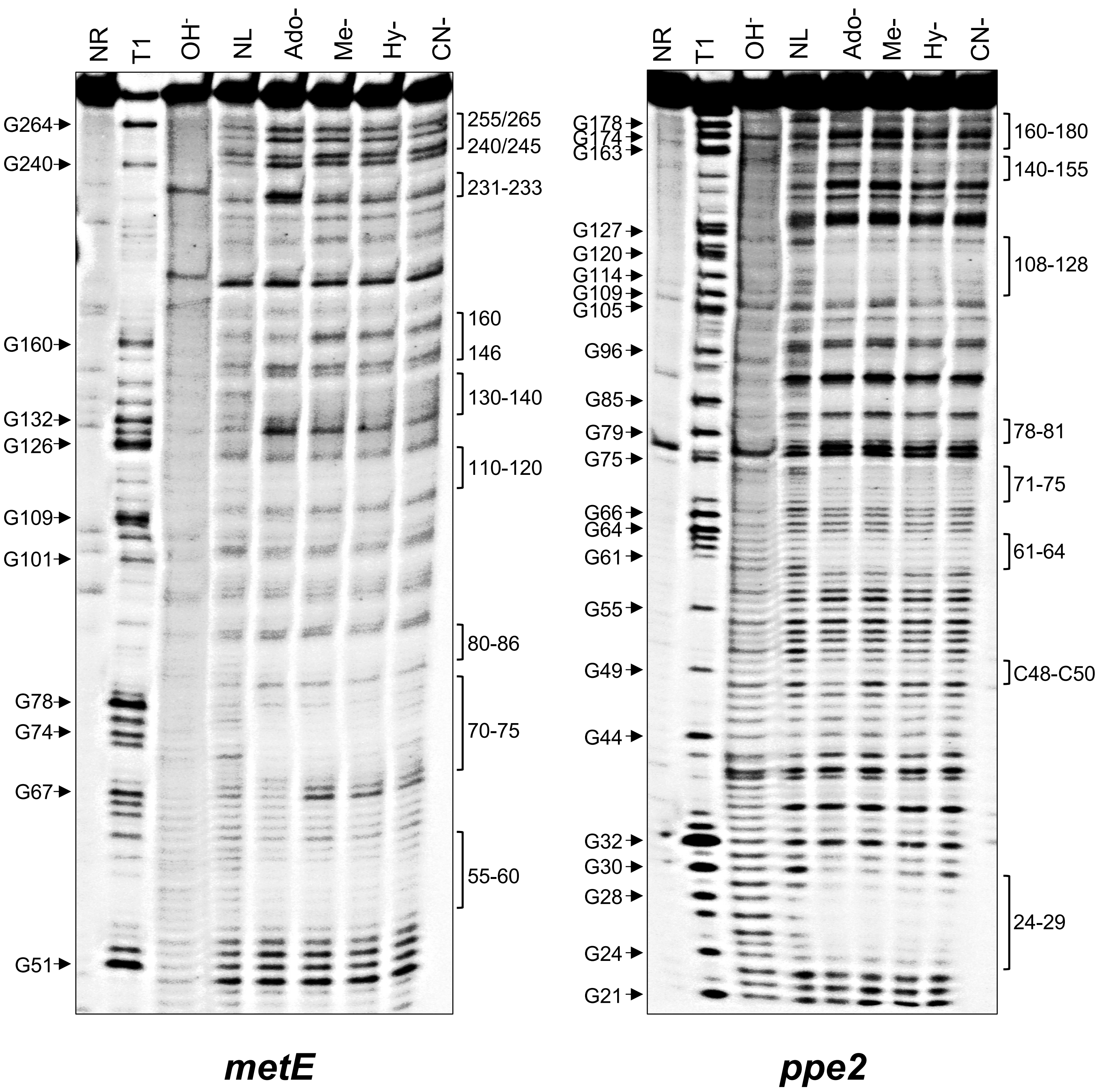

Supplementary Figure 2. Inline probing showing differences in sensitivities of *metE* and *ppe2* riboswitches to four B_{12} isoforms. Reactions contained 1 mM adenosylcobalamin (Ado-), Me- (methylcobalamin), Hy- (hydroxocobalamin), or CN- (cyanocobalamin). NR – no-reaction control; T1 – RNAse T1 ladder; OH^- – alkaline hydrolysis ladder; NL – no-ligand. Regions showing the most prominent ligand-induced modulations are indicated with brackets to the right of each gel. RNase T1-derived G positions are shown with arrows on the left of gels. The gels are cropped where no differences ± B_{12} was observed.

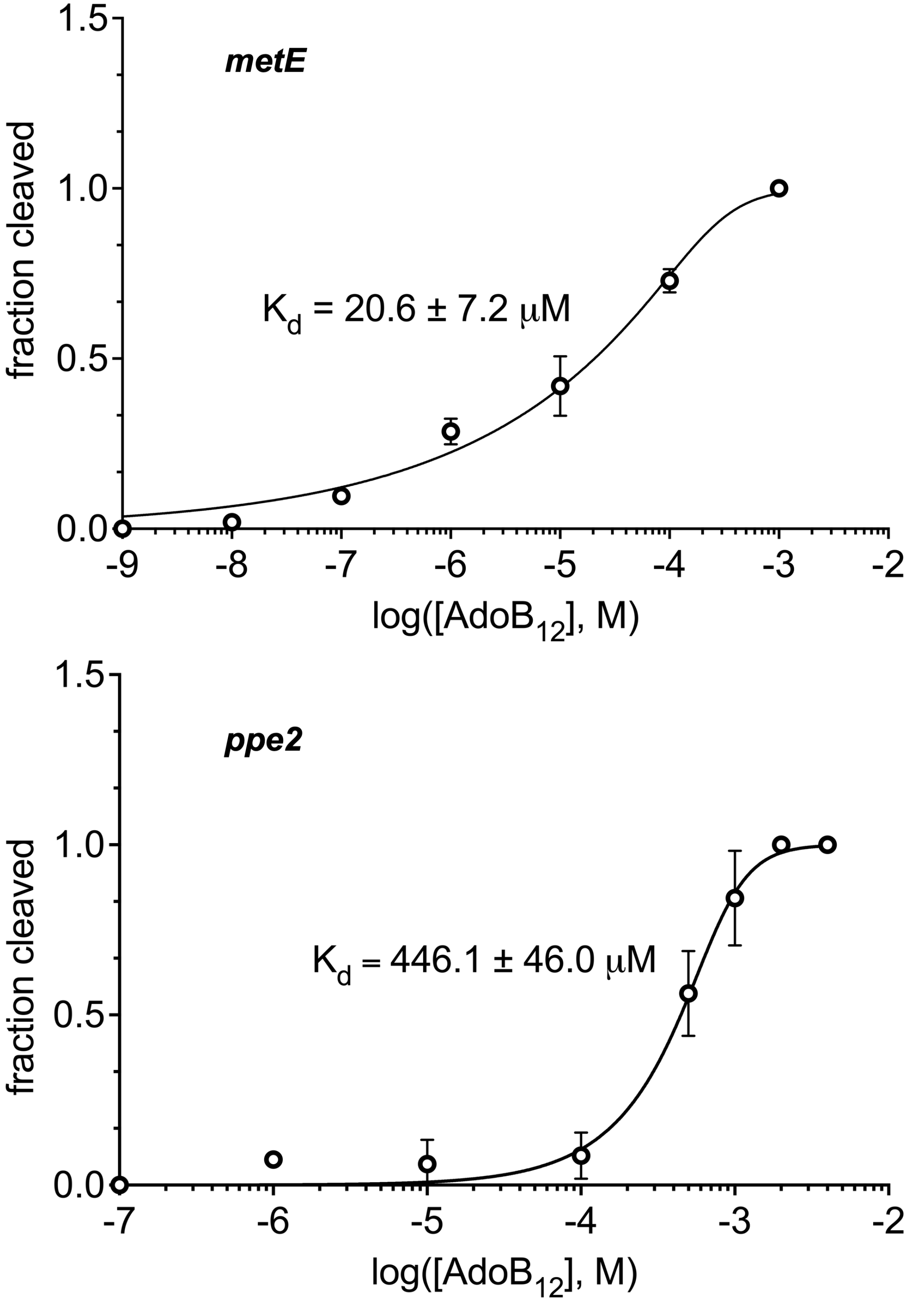

Supplementary Figure 3. $AdoB_{12}$ binding curves for *metE* and *ppe2* riboswitches. The apparent dissociation constants (K_d) of the riboswitches are calculated from the inline probing data as described in *Materials & Methods* in the main manuscript.

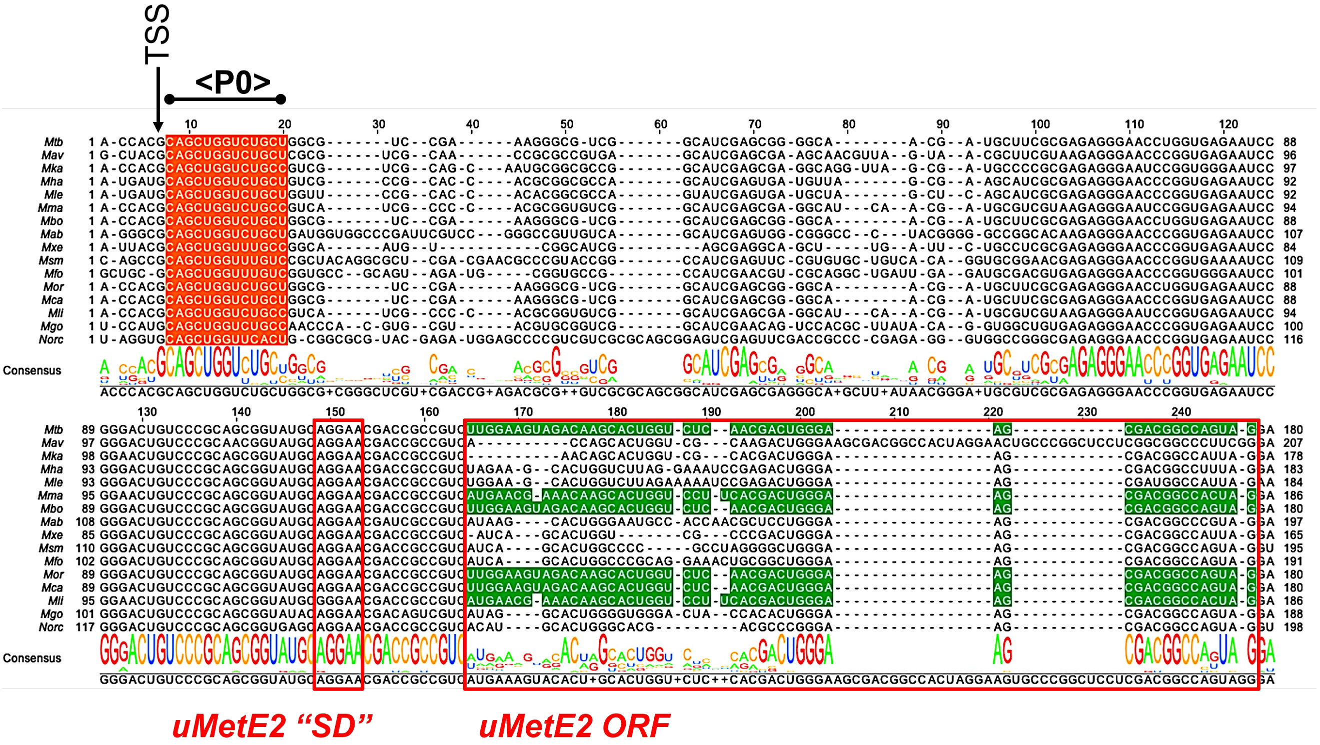

Supplementary Figure 4. Nucleic acid sequence alignment of the first 175 nt in the *metE* riboswitch. The ultra-conserved P0 element is highlighted in orange. The SD motif of uMetE2 is enclosed by a red box and the potentially productive reading frames of the uORF in different bacteria are highlighted in green and enclosed by a red border. *Mtb – M. tuberculosis; Mav – M. avium; Mka – M. kansasii; Mha – M. haemophilum; Mle – M. leprae; Mma – M. marinum; Mbo – M. bovis; Mab – M. abscessus; Mxe – M. xenopi; Msm – M. smegmatis; Mfo – M. fortuitum; Mor – M. orygis; Mca – M. canettii; Mli – M. liflandii; Mgo – M. goodii; Norc – Nocardia sp.* strain CS682.

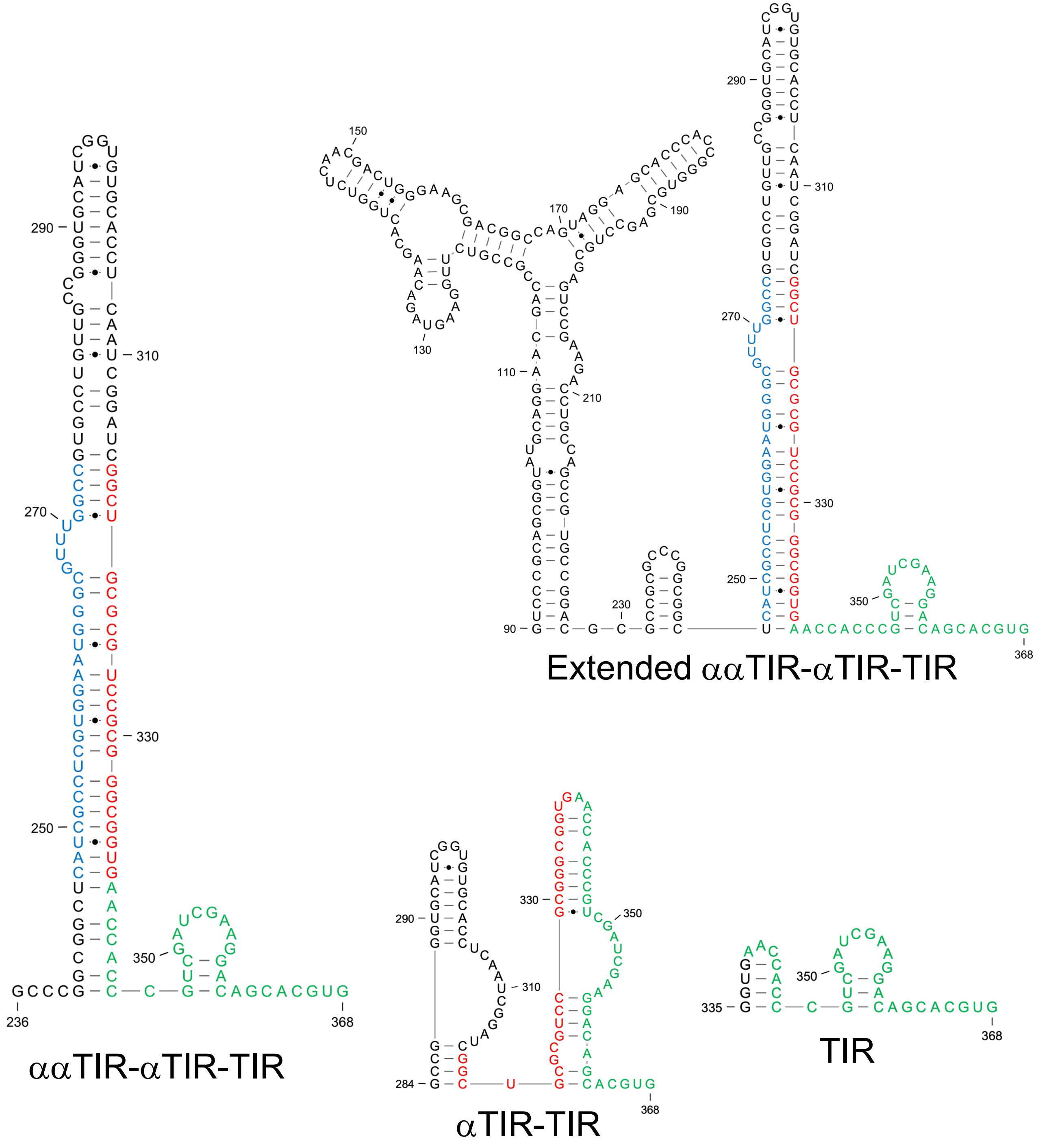

Supplementary Figure 5. Folding structures composed of highly probable base pairs for RNA segments of the *metE* leader with gradually increasing 5' edges. Translation control elements: TIR – translation initiation region; αTIR – the sequestrator of TIR; ααTIR – anti-sequestrator. The first base in each structure is numbered relative to the *metE* TSS (+1 nt).

Supplementary Figure 6

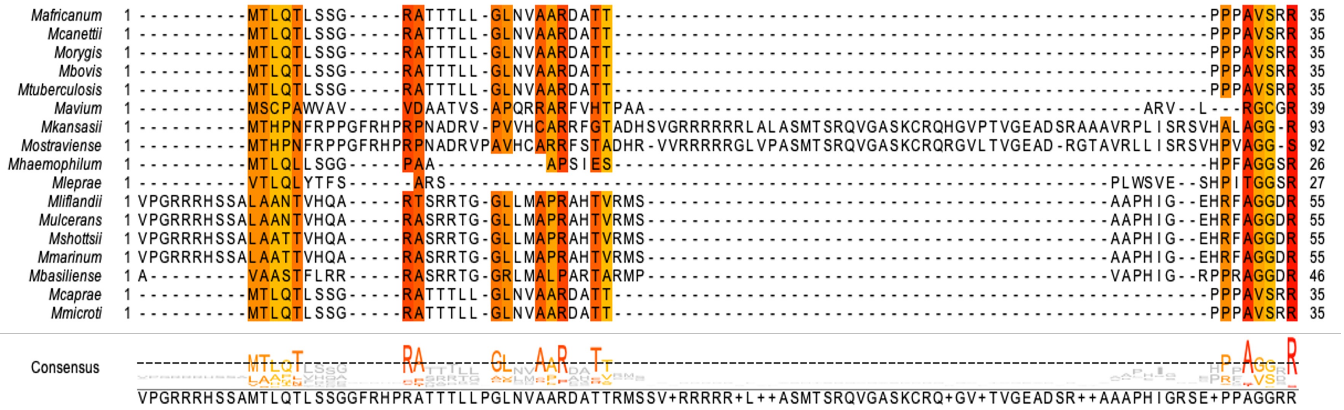

Supplementary Figure 6. Protein sequence alignment showing conservation of uPPE2 residues in representative mycobacteria. Amino acids with >50% identity (represented by the dashed threshold line across the consensus motif) are highlighted with a colour gradient (red: highest conservation; gold: lowest conservation above threshold).

Supplementary Figure 7

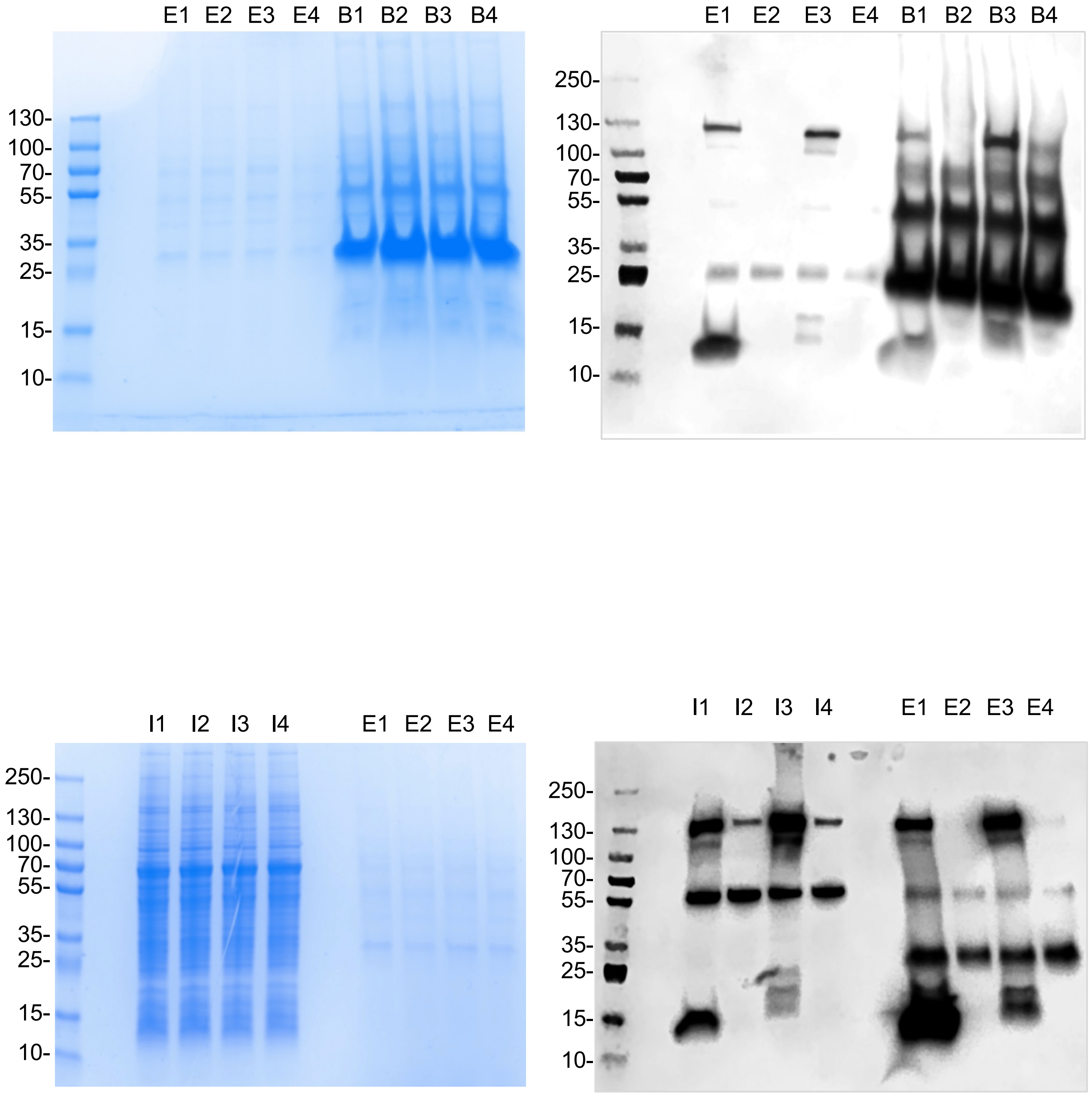

Supplementary Figure 7. Uncropped images of Coomassie gels (left) and anti-FLAG western blots (right) of FLAG-tagged and un-tagged uORF-PPE2'-LacZ constructs in multiple replicates as described in *Materials & Methods* section in the main manuscript. Lanes are indicated as either input (I), eluate (E), or bead-retained (B) fractions. Lanes: M – Page Ruler Plus, protein ladder; 1 – FLAG-uORF-PPE2'-LacZ; 2 – uORF-PPE2-LacZ; 3 – FLAG-$\text{uORF}_{\text{nostop}}$-PPE2'-LacZ; 4 – FLAG-$\text{uORF}_{\text{nostop}}$-PPE2'-LacZ.
